# Building a Framework for Peatland Assessment in Europe by Combining Spectral and Molecular Data

**DOI:** 10.1002/ece3.74658

**Published:** 2026-11-10

**Authors:** Miriam Groß‐Schmölders, Surya Gupta, Maddie Grady, Annett Wania, Fia Bengtsson, Christine Alewell

**Affiliations:** ^1^ Department of Environmental Sciences University of Basel Basel Switzerland; ^2^ Planet Labs GmbH Germany Berlin Germany; ^3^ Norwegian Institute for Nature Research Trondheim Norway

**Keywords:** hydrological disturbances, peatland degradation, peatland ecosystem functioning, plant–soil interactions, remote sensing ecology, soil molecular composition

## Abstract

Peatlands provide critical ecosystem services, including carbon storage, water regulation, and climate mitigation, yet spatial information on their ecological condition remains limited despite widespread degradation. Specific biogeochemical analyses (e.g., biomarkers, molecular compounds, compound specific stable isotopes) can characterize drainage‐related changes in peat properties but are time‐consuming, costly, linked to sophisticated analytical set‐up and thus spatially restricted. We therefore investigated whether high‐resolution PlanetScope imagery capture consistent spectral differences between pristine and drained areas and explored their multivariate correspondence with complementary site‐level biogeochemical properties. Spectral indices, including NDVI, EVI, gNDVI, Greenness Index, and albedo were derived from PlanetScope Analysis‐Ready Surface Reflectance (ARPS) data for nine European peatland sites spanning three climatic zones and contrasting open and sparsely treed vegetation structures. Peat biogeochemistry was characterized using elemental carbon and nitrogen contents and molecular composition determined by pyrolysis–GC/MS. NDVI and Greenness Index differed most consistently between the investigated pristine and drained areas, with higher values under drained conditions across both vegetation structures. In contrast, EVI and albedo, even though also suitable indicators, depended more strongly on vegetation structure and the patterns differed between open and sparsely treed systems. Spectral differences were most pronounced during May and June, identifying the early growing season as the period of strongest spectral separation within the investigated dataset. Drained areas were additionally characterized by higher nitrogen contents, lower C/N ratios, and a greater relative abundance of compounds indicative of metabolized organic matter. Exploratory principal component analyses indicated complementary site‐level patterns between spectral and biogeochemical variables associated with hydrological condition. Consequently, this study demonstrates the potential of high‐resolution PlanetScope spectral data to differentiate between pristine and drained peatland conditions across the investigated sites. However, predictive classification of previously unknown peatland areas will require dedicated modeling and independent validation especially in differentiating from surrounding transition peats and mineral grassland soils.

## Graphical abstract

This study investigates whether high‐resolution spectral vegetation indices can be used to distinguish between pristine and drained peatlands across Europe and whether these signals reflect underlying biogeochemical processes. By combining satellite‐derived indices with soil elemental and molecular data, we show that drained peatlands exhibit distinct spectral signatures linked to increased plant productivity and enhanced microbial decomposition. The results demonstrate that spectral indicators can serve as effective proxies for peatland condition and enable large‐scale ecological monitoring.

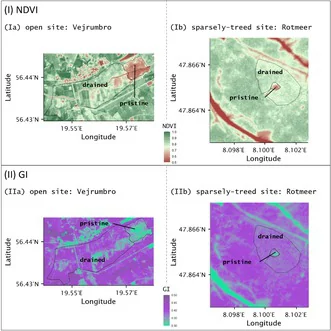

## 1 Introduction

Increasing frequency of natural extreme events as well as an increasing intensification of land use globally in the last decades leads to an intensified need to strengthen the resilience of ecosystems (Andersen et al. 2017). This is especially true for ecosystems such as pristine peatlands, which provide unique services including water filtration, carbon storage, and carbon sequestration that contribute to climate regulation and help mitigate ongoing climate change (Andersen et al. 2017). Peatland degradation is not only a loss of area but represents a fundamental shift in ecosystem functioning, altering plant community composition, nutrient cycling, and carbon dynamics (Limpens et al. 2008). However, increasing land‐use intensity and climate change have led to widespread peatland degradation. Today, only about 10% of European peatlands remain in good ecological condition (CINEA 2021). Degradation rates are particularly severe in countries such as Germany, Switzerland, the Netherlands, or Denmark, where 91%–100% of peatlands are affected (Leifeld et al. 2020; Tanneberger et al. 2021).

We started out characterizing peatland condition primarily by using biogeochemical measurements such as molecular compound composition, stable isotope analysis, and carbon and nitrogen dynamics (Groß‐Schmölders et al. 2020, 2021, 2022). Although these analyses provide detailed information on organic matter composition, nutrient status, and decomposition processes, they are time‐consuming, bound to sophisticated and high‐effort analytics, and thus costly. Their application is therefore generally restricted to a limited number of sampling locations and is difficult to extend repeatedly across large and spatially heterogeneous peatland areas. We thus started to complement these detailed field‐based measurements with remote‐sensing approaches capable of providing spatially continuous information over larger areas.

A wide range of remote sensing technologies—including optical imagery, synthetic aperture radar (SAR), LiDAR, and UAV‐based systems—have been employed to monitor vegetation dynamics, water‐table fluctuations, and land‐cover changes in peatlands (Couwenberg et al. 2020). Among optical indicators, the Normalized Difference Vegetation Index (NDVI) and Greenness Index (GI) have frequently been used to detect vegetation changes associated with moisture stress and peatland degradation (Junttila et al. 2021; McPartland et al. 2019; Tiemeyer et al. 2020; Eppinga et al. 2009; Tuittila et al. 1999, 2000). These indices reflect a combination of ecological, hydrological, and physiological processes induced by drainage. Drainage lowers the water table, increases oxygen availability in formerly anaerobic peat layers, and accelerates organic matter decomposition and nutrient mineralization (Holden et al. 2004; Räsänen et al. 2022). Reduced anoxic stress on plant roots and increased oxygen and nutrient availability can promote vascular plant growth, resulting in higher growth rates, increased leaf area index (LAI), and enhanced photosynthetic performance (Whiting 1994). Drainage also alters competitive interactions among plant functional groups and often favors grasses, shrubs, and other vascular plants over Sphagnum mosses (Rydin and Jeglum 2006). The resulting denser and taller canopies may contain more chlorophyll and consequently exhibit higher NDVI and GI values (Tuittila et al. 1999, 2000; Artz et al. 2009). NDVI and GI therefore serve as useful indicators of vegetation changes associated with peatland drainage, although their responses will also depend on vegetation structure, tree cover, seasonality, and local environmental conditions.

Recent advances in large‐scale wetland mapping, such as the updated Global Lakes and Wetlands Database (GLWD v2; Lehner et al. 2024), provide globally consistent, high‐resolution surface water information, including peatland‐relevant categories such as permanent and seasonal wetlands. However, static, long‐term representation of surface‐water occurrence does not fully capture intra‐annual variability or the dynamic hydrology of small peatland water bodies. Because peatland vegetation and hydrology exhibit strong seasonal variability, the timing of image acquisition is critical for accurately distinguishing peatland conditions. At present, there is no standardized guidance on the optimal seasonal window for remote sensing‐based peatland monitoring. This limitation, combined with the use of heterogeneous classification systems and inventory approaches (Krankina et al. 2008), limits the comparability of peatland assessment across regions and years. Consequently, spatial information on peatland condition remains fragmented and inconsistent. Addressing these limitations requires harmonized approaches that integrate multiple spectral indicators and account for complex and dynamic biophysical properties of peatland ecosystems. Although the ecological effects of drainage on peatland vegetation and soil properties are well documented, it remains uncertain whether these differences can be captured consistently by spectral indices across contrasting vegetation structures, climatic settings, and seasonal conditions. In addition, it remains unclear whether remotely sensed vegetation patterns correspond consistently with belowground biogeochemical properties at the site level.

We therefore investigated whether spectral indices consistently differentiated between pristine and drained peatland areas across contrasting vegetation structures, climatic settings, and seasonal conditions, and explored their multivariate correspondence with biogeochemical properties at the site level. We hypothesized that: (i) spectral indices show systematic differences between pristine and drained peatland areas; (ii) the magnitude and direction of these differences depend on vegetation structure and seasonality; and (iii) spectral and biogeochemical variables exhibit complementary multivariate patterns associated with hydrological condition at the site level.

## 2 Material and Methods

### 2.1 Test Sites

We investigated nine peatland sites (Figure 1, Table 1), each with a pristine and a drained part. We selected and classified these sites based on previous scientific studies in which they were identified as pristine or drained (Emsens et al. 2020; Laine et al. 1995; Lohila et al. 2010; Groß‐Schmölders et al. 2022; Ramsar Convention Secretariat 2018; Røsok 2008; Sinicyn et al. 2024; Stachowicz et al. 2025; Statsforvalteren i Trøndelag 2022). The peatlands investigated were drained between the late nineteenth century and the late twentieth century (Table 1). Drainage was primarily carried out to improve conditions for forestry and agriculture or to increase land productivity, depending on regional land‐use history. In the German Black Forest sites (Ursee, Rotmeer), drainage ditches were installed in the mid‐20th and were associated with agricultural land reclamation. Similarly, drainage in the Nordic sites mainly occurred between approximately 1900 and 1960 as part of large‐scale forestry and land improvement programs. All drained sites have therefore experienced long‐term hydrological alteration over several decades. The test sites are located across three Köppen–Geiger climate classes (Kottek et al. 2006) with four being located in the Dfc zone: Øvre Forra (Norway), Midfjellmosen (Sweden), Degerö Stormyr (Sweden) and Lakkasuo (Finland), three in the Cfb zone: Letterunshin (Ireland), Vejrumbro (Denmark), Rospuda (Poland) and two in the Dfb zone (Black Forest, Germany): Ursee and Rotmeer. The Dfc zone represents a subarctic climate with long, cold winters and short, cool summers, typically found in boreal forest regions (Peel et al. 2007). The Dfb zone is characterized as a warm‐summer humid continental climate, with cold winters and moderately warm summers—common in temperate‐continental transition zones (Peel et al. 2007; Rubel and Kottek 2010). The Cfb zone refers to a temperate oceanic climate, with mild temperatures throughout the year and no distinct dry season, typically occurring in maritime‐influenced areas such as western and central Europe (Kottek et al. 2006; Beck et al. 2018). This climatic gradient provides a valuable basis for analyzing if peatland remote sensing signals vary under different climate regimes.

**FIGURE 1:**
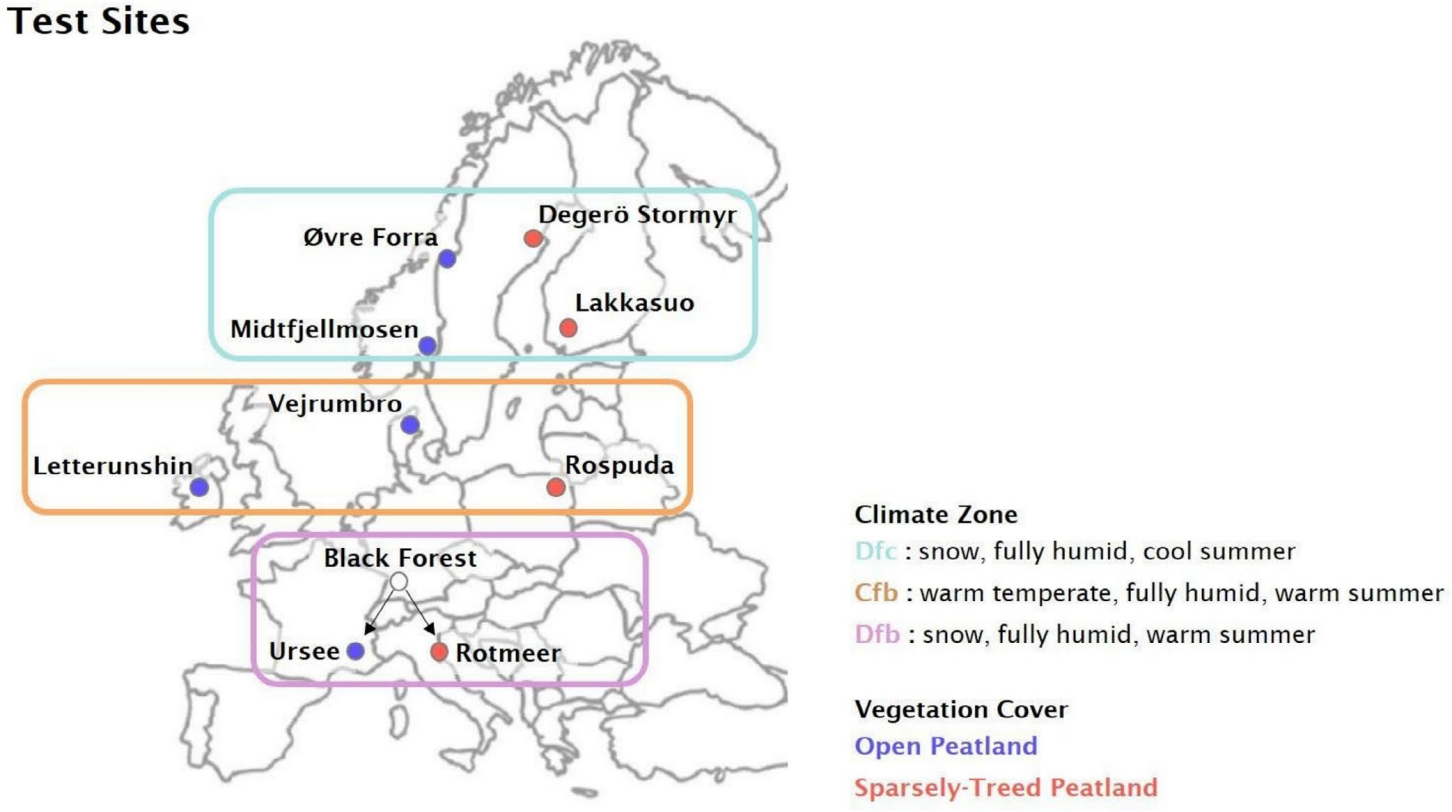
Test sites with climate zone and vegetation cover.

**TABLE 1:** Site Information for all investigated sites; MAP (Mean annual precipitation [mm]), MAT (Mean annual air temperature [°C]), WT (av. Water Table below surface [cm], natural/drained) (Emsens et al. 2020; Laine et al. 1995; Lohila et al. 2010; Groß‐Schmölders et al. 2021; Groß‐Schmölders et al. 2022; Ramsar Convention Secretariat 2018; Røsok 2008; Sinicyn et al. 2024; Stachowicz et al. 2025; Statsforvalteren i Trøndelag 2022).

Climate zone	Country	Site	Lat./Long.	Drained since	Cover type	Peatland type	MAP	MAT	WT
Dfc	Norway	Midtfjell‐mosen	59.95/11.68	~1900	Open	Bog	823	6.1	0/−21
		Øvre Forra	63.36/11.36	~1900	Open	Bog and fen	544	5.6	0/−15
	Sweden	Degerö Stormyr	64.11/19.33	~1900	Sparsely treed	Bog	523	1.2	0/−10
	Finland	Lakkasuo	61.48/24.19	1961	Sparsely treed	Bog and fen	700	3	−5/−26
Cfb	Ireland	Letterunshin	54.18/8.92	1960	Open	Bog	1.250	9.1	0/−10
	Denmark	Vejrumbro	56.44/9.55	1920	Open	Fen	678	8.2	0/−40
	Poland	Rospuda	53.8/22.6	> 1983	Sparsely treed	Fen	623	7	0/−15
Dfb	Germany	Rotmeer	47.87/8.10	~1950	Sparsely treed	Fen	1600	6	0/−15
		Ursee	48.43/8.20	~1950	Open	Fen	1400	9.5	0/−10

The peatlands included in this study were classified into two categories based on tree cover: (1) open peatlands, which lack trees, typically dominated by mosses (mainly *Sphagnum* species), graminoids ( *Eriophorum vaginatum* , *Carex rostrata* , and *Carex limosa*), and dwarf shrubs ( *Andromeda polifolia* , *Vaccinium oxycoccos* , and *Calluna vulgaris* ); and (2) sparsely treed peatlands, characterized by a low density of trees and largely visible underlying ground vegetation. Fully forested peatlands with closed or nearly closed tree canopies were outside the scope of this study. They were excluded because dense canopy cover limits the direct observation of ground vegetation and surface‐moisture conditions using optical satellite data and may cause spectral differences to reflect canopy structure rather than peatland hydrological condition (Meingast et al. 2014; Isoaho et al. 2024). We therefore focused our analysis on open peatlands (Øvre Forra, Midtfjellmosen, Letterunshin, Vejrumbro, Ursee) and sparsely treed peatlands (Degerö Stormyr, Lakkasuo, Rospuda, Rotmeer), where the underlying vegetation and surface conditions remained sufficiently visible in the PlanetScope imagery. Nevertheless, even a limited presence of trees can influence spectral indices and hydrological signals, thereby affecting the spectral signatures captured by remote sensing (Czapiewski and Szumińska 2022). Importantly, the drained areas included in this study were also restricted to open or sparsely treed peatlands.

### 2.2 Spectral Data

For this study, we used “Analysis‐Ready PlanetScope (ARPS)” multispectral satellite imagery from Planet Labs PBC averaged by month and covering the growing seasons (April–September) from 2020 to 2023. Planet offers ARPS imagery as a 3‐m orthorectified (corrected for terrain distortions) surface reflectance product (ARP‐SR) (Planet Labs, 2024). ARPS combines data from PlanetScope sensors (Planet Labs, 2023), enhancing spatial and temporal consistency, and is delivered across four spectral bands—blue, green, red, and near‐infrared (NIR).

We derived spectral indices from high‐resolution PlanetScope imagery across peatland sites spanning three climatic zones. PlanetScope data were selected over Sentinel‐2 imagery because their higher spatial resolution (3 vs. 10 m) was expected to better capture the fine‐scale spatial heterogeneity of peatland surfaces and reduce mixed‐pixel and boundary effects, particularly within relatively small and irregularly shaped study areas. In addition, the higher temporal resolution increased the availability of cloud‐free observations, which is particularly relevant under the frequently cloudy conditions of Northern Europe.

The temporal and spatial resolution makes the data well‐suited for vegetation monitoring and land cover classification (Acharki 2022; Kpienbaareh et al. 2022). To ensure data quality, each PlanetScope observation was visually inspected over the delineated pristine and drained peatland polygons. Observations were excluded entirely if snow, clouds, cloud shadows, or other visible atmospheric artifacts affected either polygon, so no additional pixel‐level masking was applied; contamination outside the delineated study polygons did not affect the extracted spectral values. PlanetScope's combination of high spatial and temporal resolution has proven effective for detecting fine‐scale land surface changes and dynamic phenomena, particularly in heterogeneous or fragmented landscapes (Arogoundade et al. 2023).

#### 2.2.1 Indices for Investigation

For our analysis, we used five spectral indices to assess the environmental conditions of the test sites: NDVI, gNDVI, GI, EVI, and albedo, according to the definitions below:

##### 2.2.1.1 The Normalized Difference Vegetation Index (NDVI)

$$\text{NDVI}=\frac{\mathrm{NIR}-\mathrm{Red}}{\mathrm{NIR}+\mathrm{Red}}$$

is a widely used spectral index for monitoring vegetation cover (Rouse et al. 1973). In peatland ecosystems, NDVI is particularly effective for assessing vegetation dynamics such as the extent of Sphagnum mosses, shrub encroachment, and rewetting effects (McPartland et al. 2019; Tuittila et al. 2000). It enables the detection of seasonal and long‐term changes in vegetation health related to hydrological variations and land‐use impacts (Tuittila et al. 2000). NDVI offers a simple yet robust measure of photosynthetic activity in peatland vegetation.

##### 2.2.1.2 The Green Normalized Difference Vegetation Index (gNDVI)

$$\text{gNDVI}=\frac{\mathrm{NIR}-\text{Green}}{\mathrm{NIR}+\text{Green}}$$

is particularly useful in peatland studies for detecting subtle differences in vegetation chlorophyll content and identifying stress in moisture‐sensitive species such as *Sphagnum* spp. (Granlund et al. 2021; Kross et al. 2016).

##### 2.2.1.3 The Greenness Index (GI)

$$\mathrm{GI}=\frac{\text{Green}}{\mathrm{Red}+\text{Green}+\text{Blue}}$$

can provide insights into vegetation greenness and structure in peatlands, especially in early successional stages or where *Sphagnum* cover dominates (Garisoain et al. 2023). Due to its reliance on the green band, GI is sensitive to changes in chlorophyll concentration and has shown potential for differentiating moss‐dominated areas from vascular plant communities (Linkosalmi et al. 2022). This makes GI useful for monitoring restoration progress or degradation in pristine or drained peatlands.

##### 2.2.1.4 The Enhanced Vegetation Index (EVI)

$$\mathrm{EVI}=2.5\;\left(\frac{\mathrm{NIR}-\mathrm{Red}}{\mathrm{NIR}+6\mathrm{Red}-7.5\text{Blue}+1}\right)$$

reduces soil background noise and atmospheric influences and thus allows a better differentiation of structurally complex vegetation, such as shrub‐invaded bogs and fen areas (Huete et al. 2002; Isoaho et al. 2024). EVI is sensitive to gross primary production, vegetation structure and composition in peatlands (Huete et al. 2002; Burdun et al. 2023).

##### 2.2.1.5 Albedo

Albedo=0.3Red+0.3NIR+0.2Blue+0.2Green

is the fraction of incoming solar radiation that is reflected by a surface, ranging from 0 (fully absorbing) to 1 (fully reflecting) (Liang 2001). Surface albedo plays a critical role in the energy balance of peatlands, which are sensitive to both climate change and hydrological disturbances (Qu et al. 2015). Albedo can serve as an indicator of different land cover types and vegetation health, including signs of bleaching or drying in peatland vegetation (Helbig, Chasmer, Kljun, et al. 2017).

### 2.3 Molecular and Biochemical Indices

Biogeochemical peat properties were characterized using stoichiometric measurements and pyrolysis gas chromatography–mass spectrometry (pyGCMS). Stoichiometry measurements provide information of bulk elemental composition sensitive to drainage‐associated changes in carbon and nitrogen dynamics (Aerts and Toet 1997), whereas pyGCMS enables the detailed characterization of molecular composition, including plant‐derived compounds, transformation products, and microbial inputs (Klein et al. 2021). These measurements were used as complementary site‐level information on peatland condition rather than as direct validation of individual spectral indices or satellite pixels.

Carbon and nitrogen contents for Midtfjellmosen and Øvre Forra were obtained from Stachowicz et al. (2025) and Ramsar Convention Secretariat (2018). For the remaining sites, soil samples were collected in 2020 and 2021 using comparable sampling protocols (Figure S1). Biogeochemical data from Degerö Stormyr, Lakkasuo, Ursee, Rotmeer, and Rospuda have been reported previously (Emsens et al. 2020; Groß‐Schmölders et al. 2020, 2021, 2022), whereas biogeochemical data from Letterunshin and Vejrumbro are presented here for the first time. At each site, soil samples were collected within homogeneous pristine and drained peatland areas to characterize local biogeochemical conditions. Three replicate peat cores were collected within each hydrological condition at Øvre Forra, Degerö Stormyr, Midtfjellmosen, Lakkasuo, Letterunshin, Vejrumbro, Ursee, and Rotmeer, and five replicate cores at Rospuda. Hydrological measurements were conducted within the same sampling area to ensure spatial correspondence with the soil observations. The sampling design was intended to characterize the biogeochemical status of each peatland condition at the plot level rather than at individual point locations. The elemental C and N contents were determined by dry combustion using a EuroEA3000 elemental analyzer. The molar carbon‐to‐nitrogen (C/N) ratios were subsequently calculated and examined.

For the molecular compound analysis, soil samples also were taken in 2020 and 2021 at the sites Vejrumbro, Letterunshin, Øvre Forra, Rospuda and Midtfjellmosen and analyzed by pyGCMS with a double‐shot pyrolizer (Frontier Laboratories, model 2020i) operating in single‐shot mode, attached to an Agilent 7890 Series GC. Approximately 1 mg of the sample was pyrolyzed for 1 min in the preheated (600°C) microfurnace. The samples were transferred with helium. The pyrolizer interface with the GC unit was held at 320°C. The GC was equipped with a 30 m column (5xi‐5sil, equivalent to HP‐5 ms) X24 μm, 0.25 μm film thickness. To improve the chromatographic separation of abundant low molecular weight molecules, the GC oven temperature was held at 37°C for 1 min and 50°C for 1 min at the start of each run (with a ramped increase of 3°Cmin^−1^ between each hold time). The oven temperature was then increased to 165°C at 3°Cmin^−1^, followed by a second increase from 165°C to 310°C at 5°Cmin^−1^, and then stabilized at 310°C for 10 min. The inlet was set to 280°C in split mode (100:1). An Agilent 5977B series mass selective detector was used to detect abundant molecules. Spectra were acquired at 70 eV ionizing energy in the 50–600 mass‐to‐charge ratio (m/z) range. Pyrolysis product identification and integration were performed using Agilent ChemStation software (2006). These more than 200 molecules were quantified in all samples and assigned by their relative abundance using the peak area of their characteristic ion fragments with a threshold cut‐off of 50 m/z. For compound assignment, mass spectra were compared with published data, as reported in the NIST 2014 mass spectral digital library, as well as with the available literature. It is worth noting that quantification is valid only for the pyrolyzed part of the organic matter (ca. 50%) that is amenable to GC but may not reflect the composition of all the organic matter present. As the Py‐GC/MS method was the same for all samples, we assumed that the pyrolytic efficiency for the different compound classes was consistent. Furthermore, the quantification indicates the relative abundances of pyrolysis products and not weight percentages. The sums of the areas of all identified peaks were set at 100%. The individual peak abundance was calculated in relative proportion to the total quantified peak area (as the relative percent abundance). The molecules were grouped according to (1) primary plant SOM and (2) metabolized SOM and microbial SOM (Table 2; Klein et al. 2021; Vancampenhout et al. 2009; Marbot 1997; Dworzanski et al. 2005). Primary plant compounds derived from fresh plant residues, such as ring monomers, amino acids, and organic acids of polysaccharides (Vancampenhout et al. 2009). Soil samples with a high proportion of primary plant compounds are characterized by a dominance of benzene over toluene and acids over aldehydes (Vancampenhout et al. 2009). These are preferentially decomposed by microbes (Vancampenhout et al. 2009; Dworzanski et al. 2005). Transformed SOM is represented by higher degraded residuals, such as low‐weight aromatics and aldehydes, as well as cyclic carbohydrates with nitrogen substitutions (Vancampenhout et al. 2009). Microbial SOM mainly features cyclic carbohydrates with nitrogen or oxygen substitutions as components of chitin and amino acids, derived from murein (Vancampenhout et al. 2009; Dworzanski et al. 2005).

**TABLE 2:** Molecules, investigated with py‐GCMS, grouped according to Klein et al. (2021), Marbot (1997), Vancampenhout et al. (2009), and Dworzanski et al. (2005).

Indicative for	Biogeochemical parameters	Identified molecules
Primary plant molecules	Polysaccharide derived from ring monomers	Levomannosan, levoglucosan, levogalactosan, dianhydrorhamnose, alpha‐delta‐glycopyranoside, sugar monomer, anhydrocyclo furanose
	Methoxy phenols (except with vinyl group)	2‐methoxy‐phenol, 4‐methoxy‐3‐methyl‐phenol, 4‐ethyl‐2‐methoxy‐phenol
	Specific N‐molecules	Methylpyridine, indole, alkylamides, nitriles, pyrazine, benzenacetonitrile
	Dominance benzene over toluene	
	Dominance acids over aldehydes	
	Organic acids	Acetic acid, propaonic acid, formic acid, citric acid, succid acid, lactic acid, butanoic acid, hexandienoic acid, butenedioic acid, carboxylic acids, benzenecarboxylic acid, carbamic acid
	Amino acids	Alanine, glutamic acid, aspartic acid, cysteine, glycine, proline, arginine, histidine, lysine, serine, threonine, tyrosine, tryptophan; glutamine, asparagine, valine, leucine, isoleucine, methionine, phenylalanine
Metabolized and microbial molecules	C12 alkene	1‐dodecene
	Low weight aromatics	Benzene, toluene, dimethylbenzene/ethylbenzene
	C2‐C6 alkyl benzenes	Styrene, p‐xylene, propyl benzene, C3‐benzene molecules, ethylbenzene‐molecules
	Low weight polysaccharides	2‐methylfuran, 2H‐furan‐3‐one, 2‐furalaldehyde, 3‐furalaldehyde, acetyl furan, 5‐methyl‐2‐furalaldehyde, 3‐hydroxy‐2‐methyl‐2‐cyclopentene‐1‐one
	Specific N‐molecules	3‐acetamidofuran, amides, pyrroles, pyridines, imidazoles, 1H‐indene, 3‐methyl‐, benzo nitrile
	Dominance of toluene over benzene	
	Specific N‐molecules	3‐acetamidofuran, amides, pyrroles, pyridines, imidazoles, 1H‐indene, 3‐methyl‐, benzo nitrile
	Absence antibacterial molecules	Nimorazole

### 2.4 ANOVA

To test the differences between pristine and drained peatland areas, a one‐way analysis of variance (ANOVA) was conducted using the R statistical software environment (R Core Team 2024) for each index. The analysis utilized monthly mean values aggregated across all sites, with data separated into pristine and drained categories. For each site and month, the mean pixel value of the corresponding area was calculated and used for statistical evaluation. ANOVA is a robust method for comparing the means of multiple groups under the assumption of normally distributed residuals and homogeneity of variances (Quinn and Keough 2002).

### 2.5 PCA

To explore the multivariate correspondence between spectral and biogeochemical variables at the site level, we performed a Principal Component Analysis (PCA) using the statistical software R (R Core Team 2024). The first PCA included spectral indices NDVI and GI and the biogeochemical variables total nitrogen content (N%), total carbon content (C%), and the C/N ratio. NDVI and GI were selected because the preceding univariate analyses showed consistent differences between pristine and drained conditions across both open and sparsely treed peatlands.

Additional PCAs were performed separately for open and sparsely treed peatlands. These analyses additionally included EVI, gNDVI, and albedo because the direction and magnitude of their responses differed between the two vegetation cover types. For each site and hydrological condition, mean values across the available observations were used.

PCA was used as an exploratory ordination method to visualize multivariate patterns and covariation among spectral and biogeochemical variables (Jolliffe 2002). The first two principal components were retained for visualization and interpretation; variable loadings were examined to assess the relative contribution of each variable to the ordination axes. PCA results were visualized using biplots generated with the factoextra (Kassambara and Mundt 2020) and ggbiplot packages of R. The spectral variables represented mean values extracted from the delineated pristine and drained polygons, whereas the biogeochemical variables represented mean values derived from replicate peat samples collected within each hydrological condition. The field measurements were used to characterize the local biogeochemical conditions of the corresponding pristine and drained peatland areas. Consequently, the field observations and spectral variables represented different spatial supports. The PCA was therefore interpreted as an exploratory site‐level comparison of complementary information and not as evidence of causal, mechanistic, or pixel‐level relationships or as pixel‐scale validation of the remote‐sensing data.

## 3 Results

### 3.1 Indices for Investigations

Our investigations revealed significant differences between pristine and drained peatland areas for NDVI (*p* < 0.001, *F* = 41.38) and GI (*p* < 0.001, *F* = 73.85) pooled over all sites (*N* = 240), with higher values in drained areas than in pristine areas (Figure 2, Figure S2). Mean monthly NDVI values ranged between 0.38 and 0.79 across the different sites under pristine conditions and increased under drained conditions (0.49 vs. 0.88). A similar trend was found for GI, which was consistently higher in drained areas (0.41 (±0.03)) compared to pristine areas (0.37 (±0.03); Figure 2, Figure S2) (Table S1).

**FIGURE 2:**
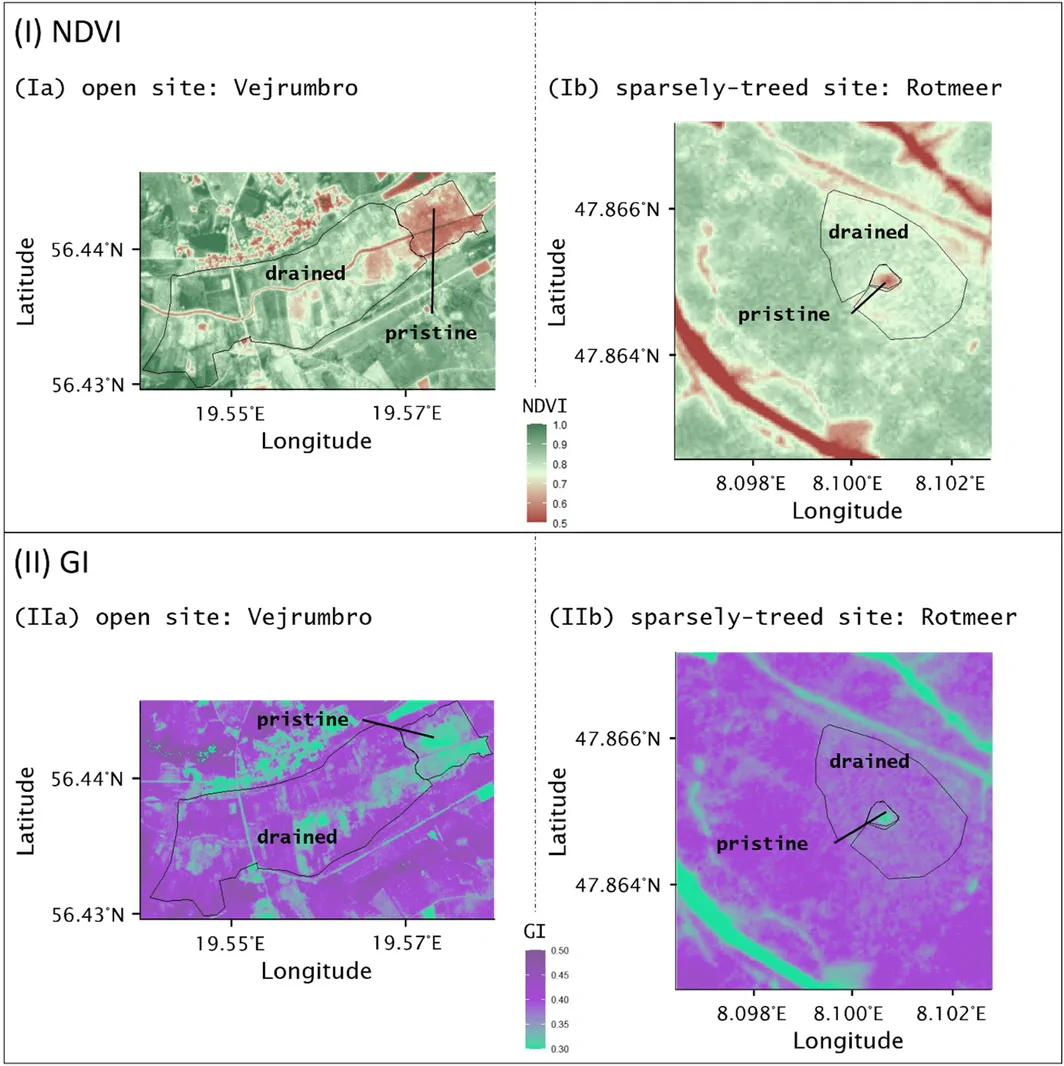
Spectral indices (I) NDVI and (II) GI for two examples: (a) open sites and (b) sparsely treed sites. Data represents a cloud‐free day in June 2023.

### 3.2 Cover Type and Their Influence of Spectral Indices

The patterns and significance varied among indices and between cover types, but not across climate zones. Three distinct response types emerged depending on the index. While NDVI and GI generally showed higher values in drained areas compared to their pristine counterparts (Figure 2, Figure S2), EVI, albedo, and gNDVI displayed cover type–dependent trends (Figure S3). For gNDVI, values were consistently higher in drained areas of sparsely treed sites (0.69–0.72) compared to pristine areas (0.67–0.69). In contrast, open sites exhibited the opposite trend, with higher values in pristine areas (0.65 ± 0.02) relative to drained ones (0.64 ± 0.02). EVI and albedo showed a reversed pattern. In open peatlands, EVI values were higher in drained areas (0.42 ± 0.08) than in pristine areas (0.35 ± 0.04), whereas in sparsely treed peatlands the opposite was true, with pristine areas showing higher values (0.35 ± 0.05) than drained ones (0.32 ± 0.04). Albedo followed the same trend: in open peatland sites values increased slightly from 0.10 ± 0.01 (pristine) to 0.11 ± 0.01 (drained), while in sparsely treed sites they decreased from 0.07 ± 0.01 (pristine) to 0.06 ± 0.01 (drained) (Table S1).

The differences for EVI and albedo were also significant between pristine and drained areas (Figure 3, Figure S3, Table 3). Only gNDVI showed no significant differences in open peatland sites (Table 3).

**FIGURE 3:**
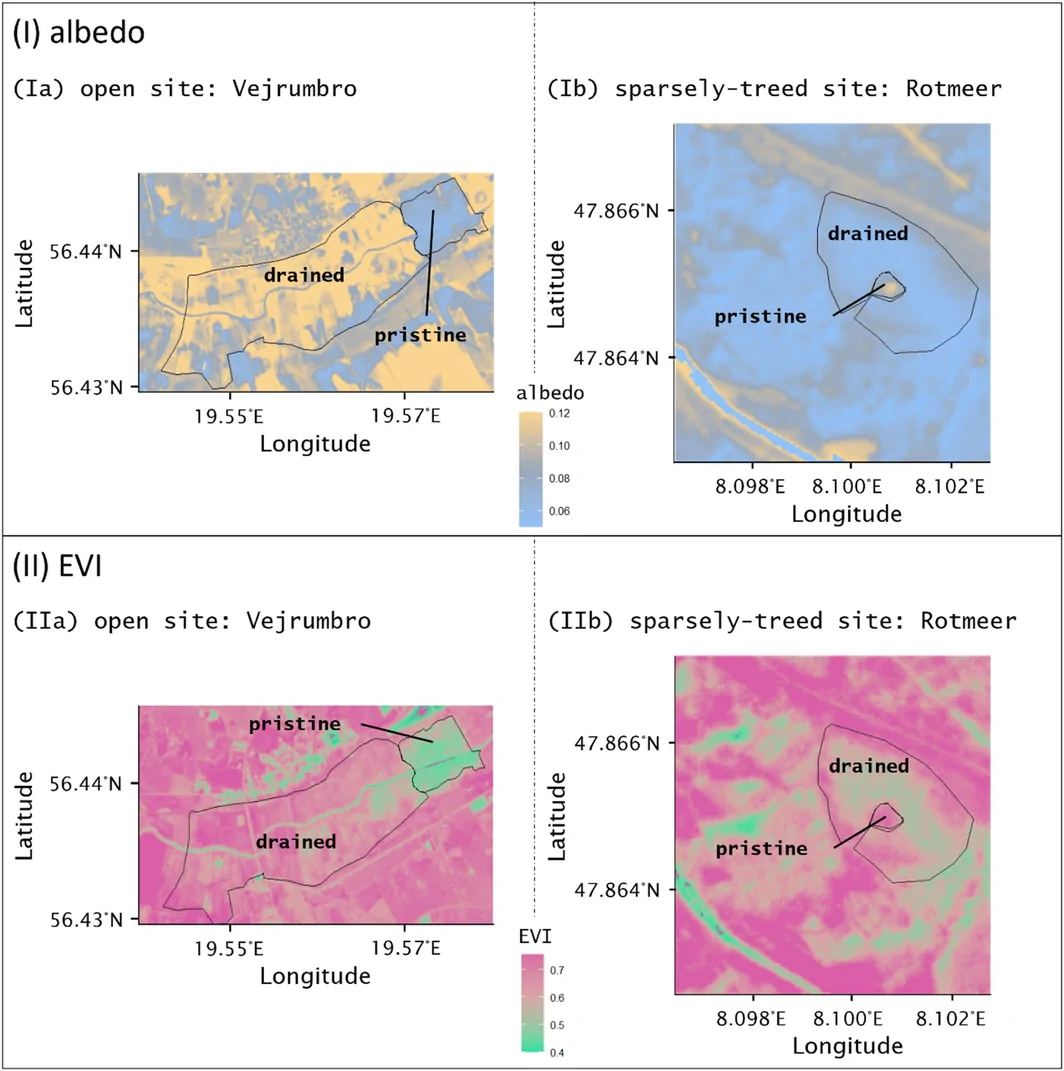
Spectral indices (I) albedo and (II) EVI for two examples: (a) open sites and (b) sparsely treed sites. Data represents a cloud‐free day in June 2023.

**TABLE 3:** *p*‐values of the ANOVA analysis (*N* = number of samples) for pristine vs. drained areas of both cover types (ST = sparsely treed): (a) spectral indices (monthly averages of all cloud‐free days, separated by period 2020–2023, spring/early summer = green, late summer/fall = beige) and (b) biogeochemical indices (molecules [M]), separated calculated for each index.

(a)																
Cover	Month	NDVI			gNDVI			GI			EVI			Albedo		
		*p*	*F*	*N*	*p*	*F*	*N*	*p*	*F*	*N*	*p*	*F*	*N*	*p*	*F*	*N*
All	All	0.001	41.38	240	0.024	1.40	240	0.001	73.85	240	0.008	2.91	240	0.005	8	240
Open	All	0.015	20.37	110	0.072	0.20	110	0.001	49.08	110	0.001	25.27	110	0.001	21.76	110
	April	0.065	3.68	30	0.893	0.02	30	0.154	2.14	30	0.019	6.23	30	0.006	8.66	30
	May	0.2	1.67	80	0.069	3.40	80	0.016	6.08	80	0.002	9.82	80	0.002	10.54	80
	June	0.016	6.01	84	0.854	0.03	84	0.001	16.37	84	0.032	4.74	84	0.426	0.66	84
	July	0.893	0.02	36	0.012	7.12	36	0.006	8.60	36	0.912	0.01	36	0.990	0.00	36
	August	0.056	3.90	40	0.027	5.32	40	0.003	10.45	40	0.167	1.98	40	0.389	0.76	40
	September	0.926	0.01	4	0.960	0.00	4	0.837	0.06	4	0.408	1.08	4	0.391	1.18	4
ST	All	0.001	25.6	130	0.001	2.28	130	0.001	27.84	130	0.001	4.73	130	0.001	129.31	130
	April	0.002	47.99	6	0.046	8.23	6	0.001	95.55	6	0.034	10.08	6	0.001	98.25	6
	May	0.001	32.91	108	0.004	8.89	108	0.000	70.17	108	0.001	12.21	108	0.000	70.09	108
	June	0.001	36.44	182	0.000	21.11	182	0.000	25.38	182	0.008	7.31	182	0.000	60.74	182
	July	0.069	7.65	88	0.198	1.68	88	0.024	5.27	88	0.013	6.41	88	0.000	31.97	88
	August	0.001	10.82	74	0.003	9.34	74	0.019	5.73	74	0.019	5.77	74	0.000	14.57	74
	September	0.021	6.40	20	0.800	0.07	20	0.130	2.51	20	0.085	3.31	20	0.000	31.34	20

(b)															
Cover	Metabolized M			Primary vs. metabolized M			C%			N%			CN ratio		
	*p*	*F*	*N*	*p*	*F*	*N*	*p*	*F*	*N*	*p*	*F*	*N*	*p*	*F*	*N*
All	0.002	10.75	98	0.001	13.54	98	0.001	1.04	214	0.001	103.31	214	0.001	133.43	214
Open	0.001	117.77	72	0.001	74.33	72	0.003	9.49	84	0.001	81.72	84	0.001	115.32	84
Sparsely treed							0.208	1.6	130	0.001	56.36	130	0.001	59.27	130

### 3.3 Seasonal Variability of Spectral Indices

Across the four‐year study period, spectral differences between pristine and drained areas varied among indices, month, and vegetation cover types (Figure 4, Figures S4–S7).

**FIGURE 4:**
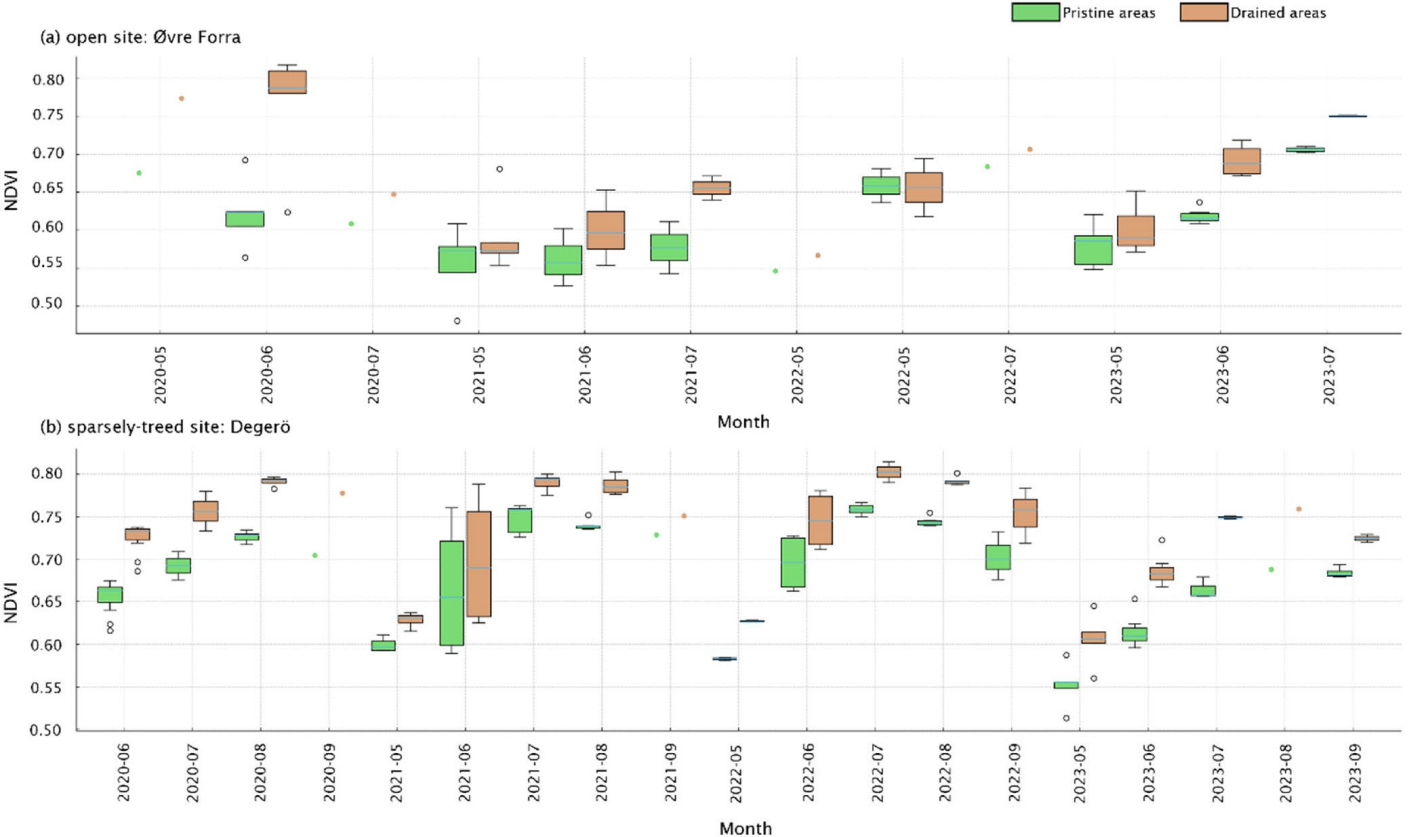
Monthly distributions of NDVI values during the growing seasons from 2020 to 2023, comparing pristine and drained areas for two peatland types: (a) open peatlands and (b) sparsely treed peatlands. Brown boxes represent drained areas, whereas green boxes represent pristine areas.

The magnitude and statistical significance of these differences varied systematically over the annual growth cycle (Figure 5, Table 3, Table S1). To determine which seasonal period showed the strongest spectral differences between pristine and drained peatlands, we compared the monthly patterns of all spectral indices. Across the investigated dataset, spectral differences were most frequently and most strongly expressed during late spring to early summer (May–June), although the pattern was not statistically significant for every index and vegetation cover type. This seasonal window therefore provided the clearest overall spectral contrast for both open and sparsely treed peatlands, highlighting this time period as particularly suitable for remote sensing–based assessments of peatland condition (Figure 5, Figures S4–S7, Table 3).

**FIGURE 5:**
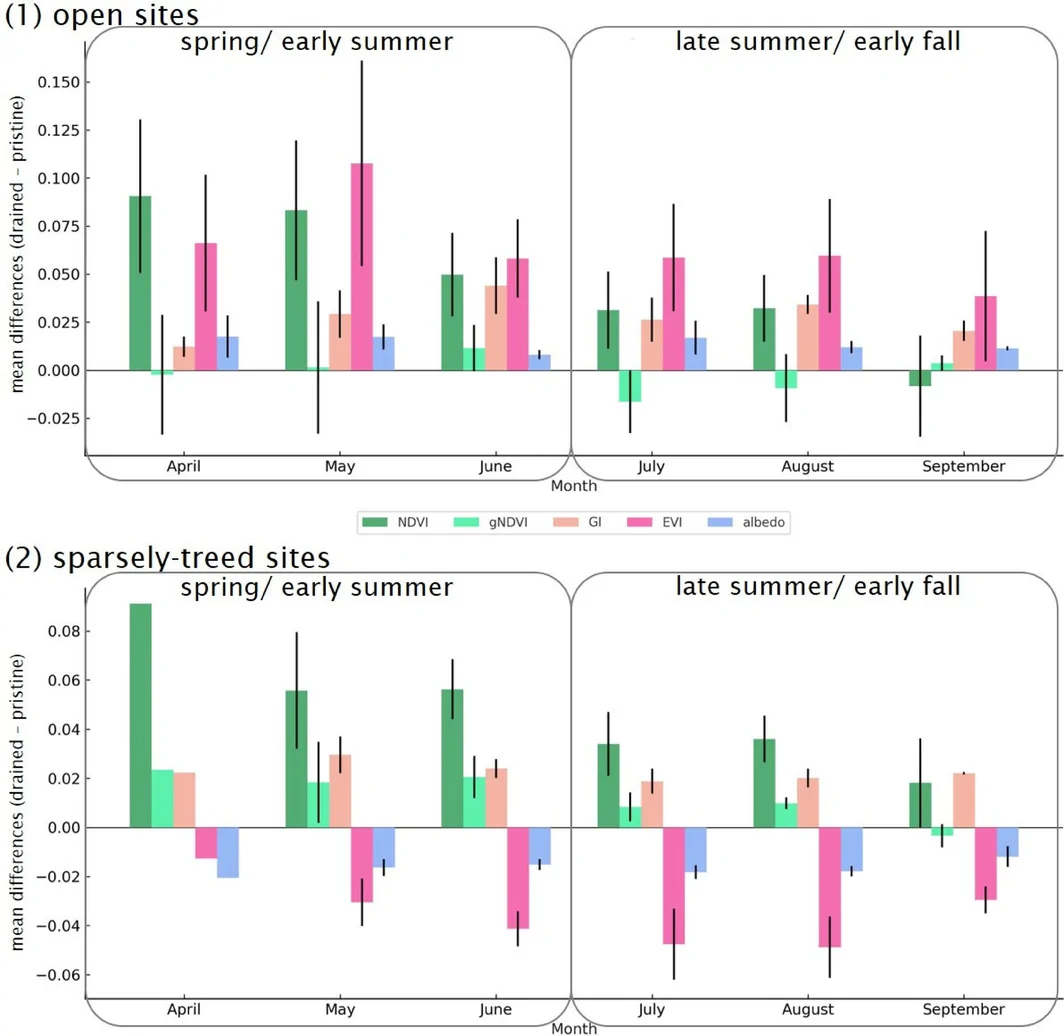
Mean monthly differences between pristine and drained areas over the period 2020–2023, separated for (1) open and (2) sparsely treed sites. For sparsely treed peatlands in April, values are based on a single site; therefore, no error bars are shown.

### 3.4 Biogeochemical Indices

#### 3.4.1 Stoichiometry

For the stoichiometry data, two of three indicators showed significant differences between pristine and drained areas. The nitrogen content was significantly higher in drained areas (1.61% ± 0.83%) compared to pristine areas (0.87% ± 0.47%; Table S2; *p* < 0.001; Table 3). This increase was accompanied by a marked reduction in the C/N ratio, with drained areas showing lower values (33.4 ± 19.0) relative to the higher ratios observed in pristine areas (52.6 (± 20.8); *p* < 0.001; Table 3).

Total carbon content did not differ significantly between the two hydrological conditions: 42.8% ± 4.1% in pristine vs. 43.1% ± 5.4% in drained areas (Table S2).

#### 3.4.2 PYGCMS

PyGCMS results revealed statistically significant differences for the molecules indicative of microbial metabolization and the ratio of molecules indicative of primary plant material to metabolized compounds (Table 3). Molecules indicative of metabolization showed a marked increase in drained areas (22.9% ± 2.0%) compared to pristine ones (12.6% ± 3.0%; *p* < 0.001; Table 3). The ratio of molecules indicative of primary plant material to metabolized compounds decreased dramatically under drainage (*p* < 0.001; Table 3), with pristine areas exhibiting a mean ratio of 3.24 ± 1.98 (Table S3) compared to 0.26 ± 0.11 in drained areas (Table S3).

### 3.5 Combination of Remote Sensing and Biogeochemical Data

The first two principal components explained a substantial portion of the overall variability, with PCA1 accounting for 67% and PCA2 for 22% of the total variance. The PCA plot showed a clear separation between pristine and drained sites. Pristine sites were generally associated with higher C/N ratios, whereas drained sites tended to align with elevated nitrogen content and lower C/N ratios. Total carbon content did not differ significantly between hydrological conditions and was therefore not interpreted as a consistent contributor to the separation between pristine and drained areas. While most observations clustered according to hydrological conditions, Letterunshin (drained) was positioned closer to the pristine observations than the other drained sites. This position indicates that Letterunshin differed from the other drained observations in the multivariate ordination; however, the exploratory PCA does not allow the underlying cause of this pattern to be determined (Figure 6).

**FIGURE 6:**
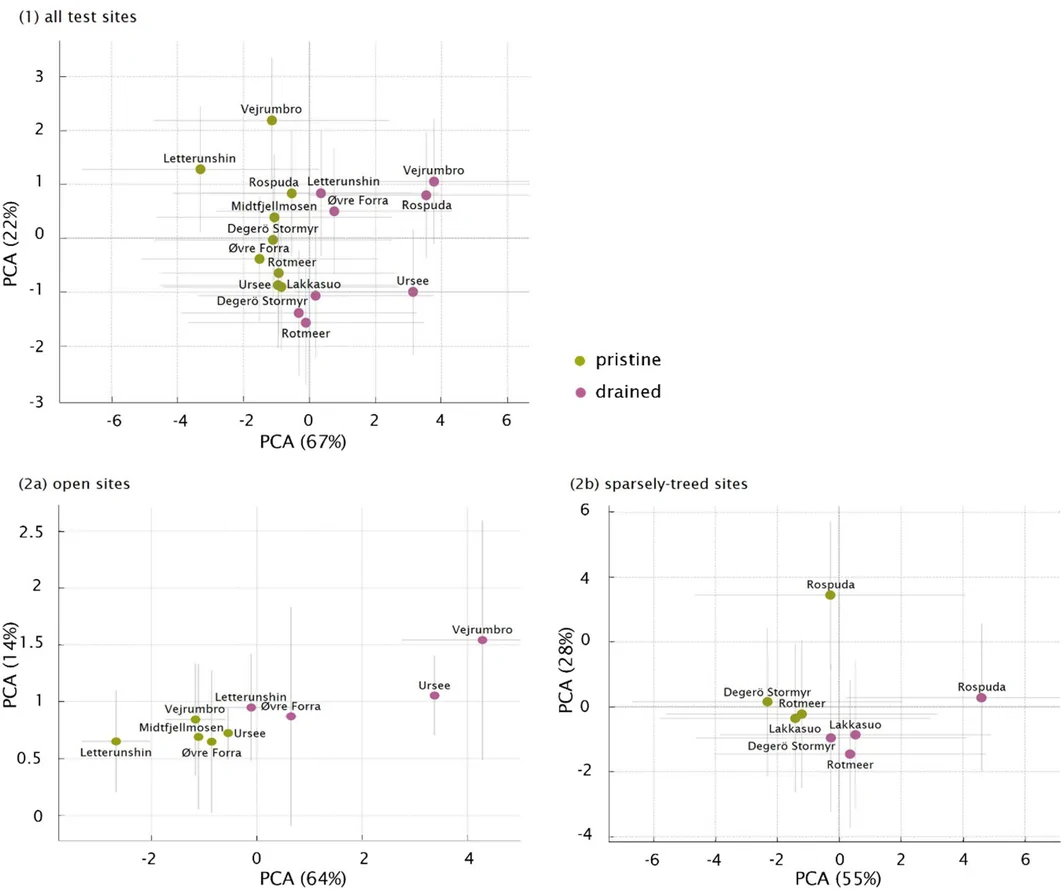
Principal component analysis (PCA) for (1) NDVI, GI, N%, C% and CN ratio for all sites and (2) additionally for EVI, gNDVI and Albedo for (2a) open and (2b) sparsely treed peatland sites (Table S4).

We conducted a second set of principal component analyses (PCA), performed separately for sparsely treed and open cover sites. In this analysis, the additional spectral indices EVI, gNDVI, and Albedo were included because they showed differences between hydrological conditions, but the direction of these varied between open and sparsely treed peatlands. For each site and hydrological condition, mean values across observations were used. For the sparsely treed cover type, the first two principal components explained 83% of the total variance (PCA1: 55%, PCA2: 28%). The PCA revealed a clear separation between drained and pristine sites. For sparsely treed peatlands, pristine observations were associated with higher EVI, albedo, and C/N ratios, whereas drained observations were associated with higher nitrogen contents and lower C/N ratios. For open cover sites, the PCA explained 78% of the total variance (PCA1: 64%, PCA2: 14%). For open peatlands, drained observations were associated with higher nitrogen contents, lower C/N ratios, and higher EVI and albedo values than pristine observations.

## 4 Discussion

### 4.1 Spectral Differences Associated With Peatland Drainage

Across all study sites—regardless of climate zone or vegetation cover—NDVI and GI values were consistently higher in drained peatland areas (Figure 2, Figure S2, Table S1). This finding reflects well‐documented ecological responses to drainage, where lowered water tables increase oxygen availability and nutrient mineralization, thereby supporting more vigorous vascular plant growth (Holden et al. 2004; Tiemeyer et al. 2020; Räsänen et al. 2022). Shifts in competitive dynamics, with increasing dominance of grasses and shrubs over *Sphagnum* mosses, result in denser, chlorophyll‐rich canopies that enhance greenness‐sensitive spectral indices (Tuittila et al. 1999, 2000; Artz et al. 2009). These patterns indicate a shift from carbon‐accumulating, nutrient‐limited systems to more nutrient‐enriched and decomposition‐driven ecosystems under drainage. Consequently, elevated NDVI and GI values represented consistent spectral indicators of drainage‐associated vegetation differences across the investigated peatland areas (Whiting 1994; Eppinga et al. 2009).

### 4.2 Differences Between Open and Sparsely Treed Peatlands

In contrast to NDVI and GI, the other spectral indices showed different patterns in open and sparsely treed sites (Figure 3, Figure S2, Table S1). These differing patterns likely reflect fundamental structural and ecological differences between the two vegetation types. For gNDVI, they showed contrasting patterns in open versus sparsely treed sites. In drained open sites, the values were higher than in pristine areas, whereas this pattern was not visible in sparsely treed sites where gNDVI values were lower. The reason for this pattern could be that in drained open areas, enhanced chlorophyll activity likely driven by improved aeration and nutrient availability in shallow‐rooted vegetation is visible (Tuittila et al. 1999), which leads to enhanced greenness and is in line with the values of NDVI and GI at these areas. In contrast, the lower values in sparsely treed drained areas, compared to pristine areas, could be due to the fact that while both NDVI and gNDVI are widely used to quantify vegetation greenness and canopy development, their spectral foundations result in differing sensitivities to environmental conditions—especially shading, which is particularly relevant in treed, drained peatlands. NDVI combines red (∼660 nm) and near‐infrared (NIR, ∼800–850 nm) reflectance to capture chlorophyll absorption and canopy structure (Tucker 1979). In contrast, gNDVI substitutes the red band with green (∼550 nm), which is only moderately absorbed by chlorophyll and more strongly affected by surface scattering and background reflectance (Gitelson et al. 1996; Gitelson and Merzlyak 1998). This makes gNDVI more sensitive to shadow effects, especially in structurally complex systems such as sparsely treed drained peatlands, where heterogeneous canopies create variable light conditions. Shadows in such environments reduce direct illumination in the green spectral range more than in the red and increase the contribution of diffuse reflectance from non‐photosynthetic elements such as branches, litter, or shaded leaves (Asner 1998). As a result, gNDVI signals may be dampened even in physiologically active vegetation, whereas NDVI remains more stable due to the stronger and more consistent absorption of red light by chlorophyll (Huete et al. 2002). Therefore, while NDVI may reflect structural biomass and greenness more robustly in such conditions, gNDVI must be interpreted cautiously, as its values may underestimate vegetation activity due to optical interference.

Regarding EVI and albedo, both show contrasting responses to drainage depending on tree cover. In open sites, drainage increased EVI and albedo, whereas in sparsely treed sites, both were lower in drained areas than in pristine ones (Figure 3, Figure S3, Table S1). Compared to NDVI, EVI reduces background and atmospheric influences by incorporating the blue spectral band and correction factors that account for soil and canopy effects (Huete et al. 2002). In drained open systems, increased vascular plant cover, higher chlorophyll content, and greater LAI lead to enhanced EVI values. In contrast, in sparsely treed drained areas, shading from tree canopies reduces the NIR—red contrast, leading to artificially lower EVI values despite high aboveground biomass (Asner 1998; Huete et al. 2002). Pristine sparsely treed peatlands with lighter canopy covers and preserved moss layers may therefore exhibit higher EVI values than drained areas.

Albedo exhibited similar tree‐cover dependent trends. Drainage decreased albedo in sparsely treed peatlands but increased in open peatlands reflecting vegetation shifts due to hydrological alteration (Zhang et al. 2014). In sparsely treed systems, a lowered water table enhances the growth of shrubs and tree saplings, increasing canopy density and reducing the proportion of exposed high‐albedo surfaces such as *Sphagnum* mosses. The resulting denser and darker canopy structure leads to a lower overall albedo (Richardson et al. 2013; Launiainen et al. 2015). In contrast, drained open peatlands are often colonized by graminoids and herbaceous species with lighter‐colored foliage and more upright canopy architecture, which can enhance reflectance, especially under high solar zenith angles (Lohila et al. 2010; Rouse et al. 1973; Zhang et al. 2014).

### 4.3 Seasonality

Across the investigated dataset, the strongest and most consistent differences between pristine and drained areas occurred in May and June, although their magnitude and statistical significance varied among spectral indices and vegetation cover types (Figure 5, Table 3). This period coincides with the early peak of the growing season, when vascular plants in drained areas exhibit rapid biomass accumulation, strong increase in leaf area index (LAI), and enhanced chlorophyll content, in contrast to the slower‐developing moss‐dominated pristine areas (Peichl et al. 2018; Rydin and Jeglum 2006; Tuittila et al. 2000; Whiting 1994). These seasonal differences in vegetation development likely contributed to the pronounced responses observed in NDVI, GI, and EVI. In contrast, albedo responses depended on vegetation structure and showed opposing patterns in open and sparsely treed peatlands (Berglund 1972; Helbig, Chasmer, Kljun, et al. 2017; Helbig, Chasmer, Desai, et al. 2017; Zhang et al. 2014). The gNDVI showed significant differences mainly in sparsely treed peatlands for April–June, whereas its response in open peatlands was less consistent. This context‐dependent pattern may reflect the sensitivity of gNDVI to canopy shadows, background reflectance, and vegetation structure (Gitelson et al. 1996). In open peatlands, statistically significant differences were restricted to fewer indices and months than in sparsely treed peatlands. This narrower and more index‐dependent response may reflect the briefer and pulse‐like growth dynamics of graminoid species and the reduced structural contrast between pristine and drained areas later in the growing season (Korrensalo et al. 2016; Weltzin et al. 2001; Peichl et al. 2018). Our results suggest that the early growing season (May–June) provides the clearest spectral separation between pristine and drained peatlands. Monitoring during the early growing season may therefore be particularly informative because it captures pronounced differences in vegetation development before canopy saturation or senescence reduces spectral contrasts (Huete et al. 2002; Asner 1998).

### 4.4 Biogeochemical Data

Our results provide clear biochemical differences between pristine and drained areas. Py‐GCMS analyses revealed a pronounced increase in the abundance of compounds indicative of microbial metabolization under drained conditions, alongside a strong decline in the ratio of primary plant‐derived to metabolized molecules (Table 3, Table S3). This pattern reflects the stimulation of microbial decomposition processes once the water table is lowered and oxygen availability increases, which accelerates organic matter breakdown (Holden et al. 2004; Andersen et al. 2017). Consistently, stoichiometric indicators demonstrated higher nitrogen contents and significantly lower C/N ratios in drained areas compared to pristine peatlands. Elevated nitrogen concentrations are characteristic of enhanced mineralization under aerobic conditions, with reduced C/N ratios pointing to a shift from carbon‐rich plant inputs toward microbially processed material (Aerts and Toet 1997; Limpens et al. 2008).

### 4.5 Complementary Patterns in Remote Sensing and Biogeochemical Data

Despite the diversity of variables examined, spectral indices and biogeochemical properties showed broadly consistent differentiation between pristine and drained peatland conditions. Drained areas were generally characterized by higher NDVI and GI values, lower C/N ratios, and a lower ratio of primary plant‐derived to metabolized compounds, whereas pristine areas showed the opposite pattern. The PCA summarized this multivariate separation and indicated that aboveground spectral characteristics and belowground biogeochemical properties covaried with hydrological condition at the site level.

These patterns are consistent with established ecological responses to drainage, including increased soil aeration, enhanced organic matter decomposition and nutrient mineralization, and the expansion of vascular vegetation (Holden et al. 2004; Tiemeyer et al. 2020; Tuittila et al. 1999; Räsänen et al. 2022). Conversely, high water tables and anaerobic soil conditions in pristine peatlands are associated with slower decomposition, lower nutrient availability, higher C/N ratios, and a greater proportion of relatively undecomposed plant‐derived material (Rydin and Jeglum 2006; Artz et al. 2009).

However, the observed correspondence should not be interpreted as evidence of direct functional or causal linkages between individual spectral and biogeochemical variables. The PCA was exploratory, and the field and remote‐sensing observations represented different spatial scales. The two data sources therefore provide complementary site‐level information on peatland conditions rather than direct biogeochemical validation of individual spectral measurements. Establishing mechanistic relationships would require spatially matched sampling and dedicated inferential or process‐based analyses.

### 4.6 Exploring the Path Forward

This study was limited to regional comparisons, balancing the need to analyze the potential of remote sensing with the limited availability of molecular data and the requirement to observe small‐scale features in remote sensing data, which calls for higher spatial detail. The main objective was to determine whether high‐resolution spectral indices showed systematic differences between the investigated pristine and drained peatland areas and whether these spectral patterns corresponded with complementary site‐level biogeochemical characteristics.

Although PlanetScope was selected to better capture fine‐scale peatland heterogeneity, the spectral indices used in this study were subsequently averaged across relatively large polygons. This spatial aggregation inevitably reduces some of the potential benefits associated with the finer spatial resolution of PlanetScope. Consequently, the present study does not demonstrate that PlanetScope provides superior performance compared with sensors offering coarser spatial resolution but higher radiometric or spectral quality, such as Sentinel‐2. However, the finer spatial resolution may still reduce the influence of mixed pixels and polygon boundaries, particularly for small, irregularly shaped or spatially heterogeneous peatland areas. A direct comparison of different satellite sensors was beyond the scope of this study and should be addressed in dedicated inter‐sensor analyses.

The contribution of the present study is thus not merely the observation that pristine and drained peatlands differ ecologically. Rather, we systematically evaluated the responses of several spectral indices across peatland sites representing contrasting vegetation structures and three climatic zones. We further assessed the seasonal consistency of these responses and identified the period during which spectral differences were most pronounced. NDVI and GI exhibited comparatively consistent differences between the investigated pristine and drained areas across open and sparsely treed peatlands, whereas EVI, gNDVI, and albedo were more dependent on vegetation structure and seasonality. The identification of May and June as the period of strongest spectral contrast provides practical information for the timing of future peatland monitoring.

The present study did not test the predictive classification of previously unknown peatland areas. Future studies could use the observed spectral patterns as predictors in dedicated classification models, which would require independent training and validation datasets and an assessment of spatial transferability. Further evaluation of the correspondence between spectral and biogeochemical indicators will also require spatially matched observations from a wider range of sites. More broadly, hydrological condition was associated with concurrent differences in vegetation characteristics and peat properties across the investigated sites.

## 5 Conclusion

This study demonstrates that high‐resolution PlanetScope ARPS data and biogeochemical indicators provide complementary site‐level information and reveal systematic differences between pristine and drained areas. NDVI and GI consistently differed between the investigated pristine and drained peatland areas across both open and sparsely treed peatlands. These patterns were observed across peatland sites representing three climatic zones (Köppen Geiger classification Dfc, Cfb, Dfb). Elevated values of NDVI and GI in drained areas were consistent with increased canopy greenness and structural development associated with hydrological alteration. In contrast, EVI, gNDVI, and albedo reflected more dynamic patterns that depended on vegetation structure and seasonality.

The early growing season—particularly May and June—provided the clearest overall spectral contrast between the pristine and drained areas within the investigated dataset, although the magnitude and statistical significance of these differences varied among indices and vegetation cover types.

PCA indicated a multivariate correspondence between spectral indices and biogeochemical properties at the site level. However, because the analysis was exploratory and the two datasets represented different spatial supports, this correspondence should not be interpreted as evidence of direct causal or functional relationships. Instead, the spectral and biogeochemical data provided complementary information on vegetation and peat properties associated with hydrological condition. Such complementary approaches may support broader assessments of peatland condition and help inform future restoration planning and monitoring.

The present study did not test the predictive classification of previously unknown peatland areas; such an application would require independent validation data and an assessment of spatial transferability.

Together, our findings demonstrate the potential of remotely sensed spectral indices to characterize peatland conditions as pristine or drained across the investigated sites, with complementary biogeochemical indicators, including C/N ratios and molecular biomarkers, providing independent site‐level support for this distinction and informing future large‐scale assessments of peatland ecosystem condition.

## Author Contributions

**Miriam Groß‐Schmölders:** conceptualization (equal). **Surya Gupta:** conceptualization (equal). **Maddie Grady:** conceptualization (equal). **Annett Wania:** conceptualization (equal). **Fia Bengtsson:** conceptualization (equal). **Christine Alewell:** conceptualization (equal).

## Funding

This work was supported by HORIZON EUROPE Climate, Energy and Mobility, 101086179.

## Conflicts of Interest

The authors declare no conflicts of interest.

## Supporting information


**Table S1:** Spectral indices for all sites, monthly.
**Table S2:** Stoichiometry data for all sites.
**Table S3:** Relative abundance of molecules [%] for all sites, separated for marker classes.
**Table S4:** Variable loadings on the first two principal components for (a) all peatland sites, (b) open peatlands, and (c) sparsely treed peatlands. Positive and negative values indicate the direction of association with each principal component, while larger absolute values indicate a stronger contribution to the respective axis.
**Figure S1:** Conceptual illustration of the sampling design in pristine and drained peatland areas. The grid represents the 3 × 3 m PlanetScope pixels, while red stars indicate the locations of three replicate peat cores collected within each hydrological condition. Spectral indices were averaged across the delineated peatland polygons, whereas biogeochemical measurements were used to characterize the sampled field plots. The illustration is schematic and not scale.
**Figure S2:** (a) NDVI and (b) GI in pristine and drained sites in early summer 2023 for all investigated sites.
**Figure S3:** (a) EVI and (b) Albedo, separated for cover types (i) open and (ii) sparsely treed, in pristine and drained sites in early summer 2023 for all investigated site.
**Figure S4:** Time series of NDVI values across all open peatland sites, distinguished between pristine and drained conditions.
**Figure S5:** Time series of GI values across all (a) open and (b) sparsely treed peatland sites, distinguished between pristine and drained conditions.
**Figure S6:** Time series of EVI values across all (a) open and (b) sparsely treed peatland sites, distinguished between pristine and drained conditions.
**Figure S7:** Time series of albedo values across all (a) open and (b) sparsely treed peatland sites, distinguished between pristine and drained conditions.

## Data Availability

Soil properties, biogeochemical data, and spectral index values are provided in the supplementary document. The PlanetScope Analysis‐Ready Surface Reflectance imagery used in this study was provided directly by Planet under a project‐specific data‐access agreement. Owing to Planet's licensing terms, the original imagery cannot be publicly redistributed by the authors. Equivalent PlanetScope ARPS data may be obtained directly from Planet under an appropriate license or data‐access agreement. The code for estimating the spectral indices is available at: https://zenodo.org/records/17897291.
