# Supplementary material for: Building a Framework for Peatland Assessment in Europe by Combining Spectral and Molecular Data
Source: Ecol Evol. 2026 Nov 10;16(11):e74658. doi: 10.1002/ece3.74658 (PMC13749602; doi:10.1002/ece3.74658)
Supplement: Supplementary file 1 [file ECE3-16-e74658-s001.docx]

Supplementary of “BUILDING A FRAMEWORK FOR PEATLAND ASSESMENT IN EUROPE BY COMBINING SPECTRAL AND MOLECULAR DATA”

**Authors**

Miriam Groß-Schmölders1(ORCID: 0000-0002-0281-4871, email: miriam.gross-schmoelders@unibas.ch)

Surya Gupta1(ORCID: 0000-0003-3643-0099, email: surya.gupta@unibas.ch)

Maddie Grady2(email: maddie.grady@planet.com)

Annett Wania2(ORCID: 0000-0001-9074-8140, email: annett@planet.com)

Fia Bengtsson3(ORCID: 0000-0003-0497-8316, email: fia.bengtsson@nina.no)

Christine Alewell1(ORCID: 0000-0001-9295-9806, email: christine.alewell@unibas.ch)

**Affiliations**

1: Department of Environmental Sciences, University of Basel, Basel, 4056, Switzerland

2: Planet Labs GmbH Germany, Kurfürstendamm 22, 10719 Berlin, Germany

3: Norwegian Institute for Nature Research, P.O. Box 5685 Torgarden, 7485 Trondheim, Norway

Content:

4 tables (S1 to S4)

7 Figures (S1 to S7)

Table S1: spectral indices for all sites, monthly

| **cover** | **site** | **month** | **NDVI** |  | **gNDVI** |  | **GI** |  | **EVI** |  | **albedo** |  |
| --- | --- | --- | --- | --- | --- | --- | --- | --- | --- | --- | --- | --- |
|  |  |  | **pristine** | **drained** | **pristine** | **drained** | **pristine** | **drained** | **pristine** | **drained** | **pristine** | **drained** |
| open | Letterunshin | 2020-05 | 0.47 | 0.67 | 0.60 | 0.60 | 0.31 | 0.42 | 0.29 | 0.40 | 0.09 | 0.12 |
| open | Letterunshin | 2020-06 | 0.55 | 0.67 | 0.61 | 0.61 | 0.34 | 0.35 | 0.36 | 0.37 | 0.10 | 0.12 |
| open | Letterunshin | 2021-05 | 0.50 | 0.69 | 0.61 | 0.61 | 0.32 | 0.38 | 0.30 | 0.38 | 0.10 | 0.12 |
| open | Letterunshin | 2021-06 | 0.59 | 0.66 | 0.66 | 0.65 | 0.34 | 0.36 | 0.37 | 0.38 | 0.10 | 0.12 |
| open | Letterunshin | 2022-04 | 0.48 | 0.66 | 0.61 | 0.62 | 0.31 | 0.41 | 0.29 | 0.42 | 0.11 | 0.11 |
| open | Letterunshin | 2022-06 | 0.58 | 0.66 | 0.66 | 0.67 | 0.33 | 0.34 | 0.34 | 0.39 | 0.10 | 0.11 |
| open | Letterunshin | 2023-05 | 0.49 | 0.67 | 0.59 | 0.64 | 0.33 | 0.38 | 0.30 | 0.38 | 0.10 | 0.11 |
| open | Letterunshin | 2023-06 | 0.51 | 0.65 | 0.60 | 0.65 | 0.33 | 0.34 | 0.31 | 0.40 | 0.11 | 0.11 |
| open | Midtfjellmosen | 2020-05 | 0.49 | 0.66 | 0.62 | 0.64 | 0.31 | 0.38 | 0.25 | 0.38 | 0.10 | 0.08 |
| open | Midtfjellmosen | 2020-06 | 0.50 | 0.62 | 0.62 | 0.63 | 0.32 | 0.37 | 0.26 | 0.38 | 0.09 | 0.10 |
| open | Midtfjellmosen | 2020-07 | 0.53 | 0.71 | 0.62 | 0.68 | 0.34 | 0.39 | 0.31 | 0.37 | 0.10 | 0.08 |
| open | Midtfjellmosen | 2020-08 | 0.53 | 0.71 | 0.62 | 0.70 | 0.34 | 0.38 | 0.29 | 0.37 | 0.09 | 0.08 |
| open | Midtfjellmosen | 2021-05 | 0.58 | 0.56 | 0.65 | 0.59 | 0.36 | 0.35 | 0.33 | 0.38 | 0.10 | 0.08 |
| open | Midtfjellmosen | 2021-06 | 0.61 | 0.68 | 0.67 | 0.66 | 0.36 | 0.39 | 0.35 | 0.36 | 0.10 | 0.10 |
| open | Midtfjellmosen | 2021-07 | 0.62 | 0.70 | 0.67 | 0.67 | 0.36 | 0.40 | 0.34 | 0.39 | 0.10 | 0.09 |
| open | Midtfjellmosen | 2021-08 | 0.65 | 0.72 | 0.68 | 0.70 | 0.38 | 0.39 | 0.34 | 0.41 | 0.09 | 0.09 |
| open | Midtfjellmosen | 2022-05 | 0.66 | 0.52 | 0.71 | 0.56 | 0.36 | 0.32 | 0.39 | 0.48 | 0.09 | 0.10 |
| open | Midtfjellmosen | 2022-06 | 0.66 | 0.63 | 0.70 | 0.64 | 0.36 | 0.38 | 0.40 | 0.37 | 0.10 | 0.10 |
| open | Midtfjellmosen | 2022-07 | 0.67 | 0.68 | 0.71 | 0.66 | 0.37 | 0.39 | 0.40 | 0.43 | 0.10 | 0.11 |
| open | Midtfjellmosen | 2022-08 | 0.67 | 0.72 | 0.72 | 0.70 | 0.36 | 0.40 | 0.40 | 0.42 | 0.09 | 0.09 |
| open | Midtfjellmosen | 2023-05 | 0.67 | 0.49 | 0.70 | 0.57 | 0.38 | 0.33 | 0.41 | 0.37 | 0.10 | 0.10 |
| open | Midtfjellmosen | 2023-06 | 0.69 | 0.55 | 0.73 | 0.59 | 0.37 | 0.35 | 0.39 | 0.36 | 0.09 | 0.11 |
| open | Ovre Forra | 2020-05 | 0.53 | 0.77 | 0.60 | 0.67 | 0.34 | 0.44 | 0.34 | 0.38 | 0.10 | 0.09 |
| open | Ovre Forra | 2020-06 | 0.54 | 0.76 | 0.64 | 0.70 | 0.34 | 0.44 | 0.32 | 0.43 | 0.11 | 0.10 |
| open | Ovre Forra | 2020-07 | 0.56 | 0.65 | 0.64 | 0.66 | 0.33 | 0.38 | 0.33 | 0.40 | 0.10 | 0.11 |
| open | Ovre Forra | 2021-05 | 0.56 | 0.56 | 0.65 | 0.57 | 0.34 | 0.44 | 0.32 | 0.36 | 0.10 | 0.11 |
| open | Ovre Forra | 2021-06 | 0.59 | 0.58 | 0.64 | 0.60 | 0.36 | 0.38 | 0.34 | 0.33 | 0.12 | 0.10 |
| open | Ovre Forra | 2021-07 | 0.60 | 0.60 | 0.66 | 0.65 | 0.35 | 0.36 | 0.38 | 0.34 | 0.11 | 0.10 |
| open | Ovre Forra | 2022-05 | 0.60 | 0.57 | 0.65 | 0.45 | 0.37 | 0.38 | 0.37 | 0.31 | 0.10 | 0.13 |
| open | Ovre Forra | 2022-06 | 0.64 | 0.66 | 0.69 | 0.64 | 0.36 | 0.38 | 0.40 | 0.41 | 0.11 | 0.11 |
| open | Ovre Forra | 2022-07 | 0.66 | 0.71 | 0.65 | 0.71 | 0.40 | 0.38 | 0.41 | 0.42 | 0.09 | 0.10 |
| open | Ovre Forra | 2023-05 | 0.66 | 0.57 | 0.70 | 0.55 | 0.38 | 0.45 | 0.34 | 0.35 | 0.11 | 0.12 |
| open | Ovre Forra | 2023-06 | 0.69 | 0.69 | 0.73 | 0.67 | 0.37 | 0.40 | 0.41 | 0.46 | 0.10 | 0.12 |
| open | Ovre Forra | 2023-07 | 0.72 | 0.75 | 0.75 | 0.73 | 0.39 | 0.41 | 0.34 | 0.49 | 0.10 | 0.11 |
| open | Ursee | 2021-04 | 0.58 | 0.71 | 0.63 | 0.57 | 0.34 | 0.40 | 0.31 | 0.33 | 0.09 | 0.09 |
| open | Ursee | 2021-05 | 0.58 | 0.76 | 0.64 | 0.62 | 0.33 | 0.46 | 0.32 | 0.57 | 0.09 | 0.12 |
| open | Ursee | 2021-09 | 0.60 | 0.87 | 0.66 | 0.82 | 0.34 | 0.49 | 0.31 | 0.54 | 0.09 | 0.09 |
| open | Ursee | 2022-04 | 0.62 | 0.70 | 0.58 | 0.62 | 0.38 | 0.40 | 0.31 | 0.44 | 0.08 | 0.11 |
| open | Ursee | 2022-06 | 0.71 | 0.88 | 0.70 | 0.78 | 0.39 | 0.56 | 0.39 | 0.64 | 0.12 | 0.12 |
| open | Ursee | 2022-07 | 0.72 | 0.82 | 0.72 | 0.75 | 0.39 | 0.50 | 0.43 | 0.61 | 0.11 | 0.13 |
| open | Ursee | 2022-08 | 0.63 | 0.79 | 0.74 | 0.75 | 0.38 | 0.48 | 0.46 | 0.55 | 0.10 | 0.12 |
| open | Ursee | 2023-04 | 0.74 | 0.71 | 0.73 | 0.58 | 0.37 | 0.39 | 0.41 | 0.30 | 0.11 | 0.11 |
| open | Ursee | 2023-05 | 0.75 | 0.73 | 0.23 | 0.57 | 0.33 | 0.43 | 0.44 | 0.47 | 0.09 | 0.10 |
| open | Ursee | 2023-06 | 0.73 | 0.82 | 0.73 | 0.74 | 0.41 | 0.50 | 0.47 | 0.61 | 0.10 | 0.12 |
| open | Ursee | 2023-08 | 0.75 | 0.81 | 0.81 | 0.76 | 0.43 | 0.47 | 0.44 | 0.61 | 0.08 | 0.12 |
| open | Vejumbro | 2020-04 | 0.38 | 0.50 | 0.51 | 0.58 | 0.32 | 0.44 | 0.19 | 0.32 | 0.09 | 0.12 |
| open | Vejumbro | 2020-08 | 0.38 | 0.79 | 0.51 | 0.74 | 0.32 | 0.43 | 0.19 | 0.56 | 0.09 | 0.12 |
| open | Vejumbro | 2021-04 | 0.39 | 0.54 | 0.52 | 0.54 | 0.31 | 0.43 | 0.18 | 0.29 | 0.08 | 0.13 |
| open | Vejumbro | 2021-05 | 0.53 | 0.61 | 0.57 | 0.60 | 0.36 | 0.40 | 0.30 | 0.45 | 0.10 | 0.14 |
| open | Vejumbro | 2022-05 | 0.45 | 0.87 | 0.52 | 0.74 | 0.34 | 0.49 | 0.23 | 0.77 | 0.09 | 0.14 |
| open | Vejumbro | 2022-07 | 0.72 | 0.67 | 0.69 | 0.65 | 0.41 | 0.42 | 0.46 | 0.55 | 0.11 | 0.15 |
| open | Vejumbro | 2022-09 | 0.77 | 0.74 | 0.73 | 0.73 | 0.42 | 0.40 | 0.47 | 0.47 | 0.10 | 0.11 |
| open | Vejumbro | 2023-04 | 0.40 | 0.55 | 0.54 | 0.61 | 0.31 | 0.45 | 0.18 | 0.34 | 0.08 | 0.11 |
| open | Vejumbro | 2023-05 | 0.56 | 0.73 | 0.58 | 0.69 | 0.37 | 0.41 | 0.32 | 0.53 | 0.10 | 0.13 |
| open | Vejumbro | 2023-06 | 0.79 | 0.79 | 0.72 | 0.74 | 0.35 | 0.43 | 0.54 | 0.59 | 0.11 | 0.13 |
| ST | Lakkasuo | 2020-05 | 0.55 | 0.66 | 0.64 | 0.66 | 0.34 | 0.47 | 0.30 | 0.30 | 0.09 | 0.07 |
| ST | Lakkasuo | 2020-06 | 0.64 | 0.74 | 0.67 | 0.70 | 0.37 | 0.41 | 0.39 | 0.37 | 0.10 | 0.08 |
| ST | Lakkasuo | 2020-07 | 0.70 | 0.81 | 0.74 | 0.75 | 0.40 | 0.43 | 0.47 | 0.42 | 0.10 | 0.08 |
| ST | Lakkasuo | 2020-08 | 0.71 | 0.83 | 0.75 | 0.77 | 0.40 | 0.44 | 0.45 | 0.41 | 0.09 | 0.07 |
| ST | Lakkasuo | 2021-05 | 0.59 | 0.66 | 0.63 | 0.66 | 0.36 | 0.34 | 0.31 | 0.30 | 0.08 | 0.07 |
| ST | Lakkasuo | 2021-06 | 0.65 | 0.72 | 0.67 | 0.69 | 0.39 | 0.40 | 0.42 | 0.37 | 0.10 | 0.08 |
| ST | Lakkasuo | 2021-07 | 0.76 | 0.79 | 0.74 | 0.74 | 0.41 | 0.43 | 0.48 | 0.41 | 0.10 | 0.08 |
| ST | Lakkasuo | 2021-08 | 0.74 | 0.80 | 0.76 | 0.75 | 0.41 | 0.42 | 0.46 | 0.39 | 0.09 | 0.07 |
| ST | Lakkasuo | 2021-09 | 0.72 | 0.77 | 0.76 | 0.75 | 0.36 | 0.39 | 0.38 | 0.35 | 0.08 | 0.07 |
| ST | Lakkasuo | 2022-05 | 0.54 | 0.60 | 0.60 | 0.61 | 0.34 | 0.39 | 0.32 | 0.30 | 0.10 | 0.09 |
| ST | Lakkasuo | 2022-06 | 0.70 | 0.71 | 0.67 | 0.69 | 0.39 | 0.40 | 0.44 | 0.36 | 0.10 | 0.08 |
| ST | Lakkasuo | 2022-07 | 0.73 | 0.72 | 0.72 | 0.69 | 0.41 | 0.41 | 0.48 | 0.40 | 0.11 | 0.09 |
| ST | Lakkasuo | 2022-08 | 0.74 | 0.78 | 0.76 | 0.74 | 0.40 | 0.41 | 0.50 | 0.42 | 0.10 | 0.08 |
| ST | Lakkasuo | 2022-09 | 0.71 | 0.74 | 0.74 | 0.72 | 0.36 | 0.38 | 0.42 | 0.36 | 0.09 | 0.07 |
| ST | Lakkasuo | 2023-05 | 0.57 | 0.62 | 0.63 | 0.65 | 0.34 | 0.36 | 0.32 | 0.30 | 0.09 | 0.07 |
| ST | Lakkasuo | 2023-06 | 0.65 | 0.69 | 0.67 | 0.68 | 0.38 | 0.39 | 0.42 | 0.38 | 0.11 | 0.07 |
| ST | Lakkasuo | 2023-07 | 0.75 | 0.78 | 0.74 | 0.73 | 0.40 | 0.40 | 0.50 | 0.44 | 0.10 | 0.08 |
| ST | Lakkasuo | 2023-08 | 0.76 | 0.78 | 0.74 | 0.73 | 0.40 | 0.40 | 0.50 | 0.40 | 0.10 | 0.08 |
| ST | Lakkasuo | 2023-09 | 0.71 | 0.77 | 0.73 | 0.73 | 0.37 | 0.43 | 0.41 | 0.38 | 0.09 | 0.07 |
| ST | Rospuda | 2020-05 | 0.49 | 0.58 | 0.54 | 0.59 | 0.32 | 0.36 | 0.29 | 0.28 | 0.09 | 0.06 |
| ST | Rospuda | 2020-06 | 0.66 | 0.76 | 0.64 | 0.69 | 0.39 | 0.43 | 0.42 | 0.42 | 0.08 | 0.05 |
| ST | Rospuda | 2020-07 | 0.64 | 0.88 | 0.76 | 0.78 | 0.40 | 0.50 | 0.69 | 0.57 | 0.08 | 0.05 |
| ST | Rospuda | 2020-08 | 0.67 | 0.87 | 0.77 | 0.78 | 0.40 | 0.50 | 0.66 | 0.55 | 0.08 | 0.05 |
| ST | Rospuda | 2021-05 | 0.51 | 0.62 | 0.56 | 0.63 | 0.35 | 0.36 | 0.29 | 0.28 | 0.08 | 0.05 |
| ST | Rospuda | 2021-06 | 0.68 | 0.85 | 0.72 | 0.75 | 0.33 | 0.47 | 0.58 | 0.53 | 0.08 | 0.06 |
| ST | Rospuda | 2021-08 | 0.65 | 0.86 | 0.76 | 0.77 | 0.41 | 0.46 | 0.62 | 0.54 | 0.10 | 0.05 |
| ST | Rospuda | 2022-05 | 0.43 | 0.57 | 0.53 | 0.60 | 0.37 | 0.35 | 0.28 | 0.24 | 0.08 | 0.06 |
| ST | Rospuda | 2022-06 | 0.63 | 0.81 | 0.68 | 0.71 | 0.42 | 0.45 | 0.51 | 0.48 | 0.09 | 0.06 |
| ST | Rospuda | 2022-07 | 0.62 | 0.86 | 0.76 | 0.77 | 0.34 | 0.48 | 0.66 | 0.58 | 0.08 | 0.06 |
| ST | Rospuda | 2022-08 | 0.63 | 0.85 | 0.75 | 0.76 | 0.43 | 0.47 | 0.60 | 0.53 | 0.11 | 0.05 |
| ST | Rospuda | 2023-05 | 0.41 | 0.55 | 0.52 | 0.59 | 0.43 | 0.35 | 0.28 | 0.25 | 0.09 | 0.06 |
| ST | Rospuda | 2023-06 | 0.64 | 0.77 | 0.64 | 0.70 | 0.38 | 0.42 | 0.49 | 0.45 | 0.09 | 0.07 |
| ST | Rospuda | 2023-07 | 0.61 | 0.84 | 0.73 | 0.74 | 0.41 | 0.47 | 0.60 | 0.57 | 0.08 | 0.06 |
| ST | Rotmeer | 2020-04 | 0.56 | 0.67 | 0.64 | 0.67 | 0.33 | 0.36 | 0.28 | 0.28 | 0.09 | 0.06 |
| ST | Rotmeer | 2020-07 | 0.69 | 0.72 | 0.65 | 0.66 | 0.39 | 0.39 | 0.42 | 0.37 | 0.10 | 0.08 |
| ST | Rotmeer | 2020-08 | 0.70 | 0.73 | 0.69 | 0.69 | 0.37 | 0.39 | 0.40 | 0.36 | 0.09 | 0.07 |
| ST | Rotmeer | 2021-04 | 0.60 | 0.67 | 0.66 | 0.68 | 0.33 | 0.35 | 0.29 | 0.28 | 0.08 | 0.06 |
| ST | Rotmeer | 2021-05 | 0.71 | 0.71 | 0.66 | 0.65 | 0.32 | 0.41 | 0.37 | 0.31 | 0.08 | 0.07 |
| ST | Rotmeer | 2021-09 | 0.65 | 0.63 | 0.64 | 0.63 | 0.42 | 0.44 | 0.38 | 0.36 | 0.10 | 0.10 |
| ST | Rotmeer | 2022-04 | 0.60 | 0.69 | 0.65 | 0.67 | 0.34 | 0.36 | 0.30 | 0.28 | 0.09 | 0.06 |
| ST | Rotmeer | 2022-06 | 0.75 | 0.81 | 0.73 | 0.75 | 0.41 | 0.44 | 0.41 | 0.37 | 0.09 | 0.07 |
| ST | Rotmeer | 2022-07 | 0.69 | 0.79 | 0.69 | 0.73 | 0.38 | 0.42 | 0.43 | 0.39 | 0.10 | 0.07 |
| ST | Rotmeer | 2022-08 | 0.69 | 0.79 | 0.70 | 0.74 | 0.37 | 0.42 | 0.39 | 0.36 | 0.09 | 0.07 |
| ST | Rotmeer | 2023-05 | 0.64 | 0.65 | 0.64 | 0.63 | 0.36 | 0.37 | 0.35 | 0.29 | 0.09 | 0.07 |
| ST | Rotmeer | 2023-06 | 0.74 | 0.75 | 0.69 | 0.69 | 0.39 | 0.40 | 0.44 | 0.38 | 0.10 | 0.08 |
| ST | Rotmeer | 2023-08 | 0.78 | 0.80 | 0.75 | 0.75 | 0.39 | 0.39 | 0.47 | 0.39 | 0.09 | 0.07 |
| ST | Degerö | 2020-06 | 0.66 | 0.70 | 0.68 | 0.71 | 0.37 | 0.40 | 0.39 | 0.40 | 0.10 | 0.08 |
| ST | Degerö | 2020-07 | 0.69 | 0.74 | 0.71 | 0.73 | 0.37 | 0.40 | 0.42 | 0.44 | 0.10 | 0.08 |
| ST | Degerö | 2020-08 | 0.73 | 0.71 | 0.74 | 0.76 | 0.38 | 0.42 | 0.45 | 0.46 | 0.10 | 0.08 |
| ST | Degerö | 2020-09 | 0.70 | 0.76 | 0.74 | 0.76 | 0.37 | 0.40 | 0.42 | 0.44 | 0.10 | 0.09 |
| ST | Degerö | 2021-05 | 0.60 | 0.61 | 0.66 | 0.66 | 0.34 | 0.35 | 0.30 | 0.30 | 0.08 | 0.08 |
| ST | Degerö | 2021-06 | 0.66 | 0.68 | 0.69 | 0.69 | 0.37 | 0.39 | 0.38 | 0.37 | 0.09 | 0.08 |
| ST | Degerö | 2021-07 | 0.75 | 0.77 | 0.74 | 0.75 | 0.40 | 0.43 | 0.48 | 0.47 | 0.11 | 0.09 |
| ST | Degerö | 2021-08 | 0.74 | 0.73 | 0.75 | 0.75 | 0.38 | 0.41 | 0.46 | 0.45 | 0.10 | 0.09 |
| ST | Degerö | 2021-09 | 0.73 | 0.73 | 0.76 | 0.75 | 0.37 | 0.39 | 0.42 | 0.40 | 0.09 | 0.08 |
| ST | Degerö | 2022-05 | 0.58 | 0.61 | 0.68 | 0.68 | 0.32 | 0.35 | 0.31 | 0.30 | 0.09 | 0.08 |
| ST | Degerö | 2022-06 | 0.70 | 0.73 | 0.69 | 0.73 | 0.38 | 0.41 | 0.40 | 0.40 | 0.09 | 0.08 |
| ST | Degerö | 2022-07 | 0.76 | 0.77 | 0.74 | 0.75 | 0.40 | 0.44 | 0.49 | 0.48 | 0.11 | 0.08 |
| ST | Degerö | 2022-08 | 0.74 | 0.77 | 0.76 | 0.77 | 0.39 | 0.42 | 0.47 | 0.45 | 0.11 | 0.08 |
| ST | Degerö | 2022-09 | 0.70 | 0.73 | 0.76 | 0.77 | 0.35 | 0.38 | 0.43 | 0.40 | 0.10 | 0.08 |
| ST | Degerö | 2023-05 | 0.55 | 0.59 | 0.64 | 0.64 | 0.33 | 0.35 | 0.37 | 0.32 | 0.10 | 0.09 |
| ST | Degerö | 2023-06 | 0.61 | 0.67 | 0.67 | 0.69 | 0.34 | 0.38 | 0.38 | 0.38 | 0.11 | 0.09 |
| ST | Degerö | 2023-07 | 0.66 | 0.73 | 0.70 | 0.73 | 0.36 | 0.39 | 0.44 | 0.45 | 0.12 | 0.09 |
| ST | Degerö | 2023-08 | 0.69 | 0.74 | 0.70 | 0.73 | 0.37 | 0.40 | 0.46 | 0.46 | 0.12 | 0.08 |
| ST | Degerö | 2023-09 | 0.68 | 0.70 | 0.72 | 0.73 | 0.34 | 0.35 | 0.41 | 0.38 | 0.10 | 0.08 |

Table S2: Stoichiometry data for all sites

| **cover** | **site** | **sample_no** | **N [%]** |  | **C [%]** |  | **C/N ratio** | |
| --- | --- | --- | --- | --- | --- | --- | --- | --- |
|  |  |  | **pristine** | **drained** | **pristine** | **drained** | **pristine** | **drained** |
| open | Letterunshin | 1 | 0.63 | 0.98 | 44.66 | 40.37 | 65.53 | 45.98 |
| open | Letterunshin | 2 | 0.63 | 1.08 | 44.34 | 43.66 | 60.68 | 45.22 |
| open | Letterunshin | 3 | 0.60 | 1.08 | 44.39 | 43.80 | 62.93 | 45.37 |
| open | Letterunshin | 4 | 0.73 | 0.96 | 45.25 | 35.27 | 53.49 | 40.68 |
| open | Letterunshin | 5 | 0.60 | 1.07 | 44.43 | 44.75 | 63.52 | 46.65 |
| open | Letterunshin | 6 | 0.67 | 1.07 | 44.81 | 34.77 | 58.56 | 35.98 |
| open | Letterunshin | 7 | 0.61 | 1.01 | 44.49 | 40.52 | 63.25 | 44.62 |
| open | Letterunshin | 8 | 0.62 | 1.10 | 44.54 | 42.96 | 61.57 | 43.69 |
| open | Ovre Forra | 1 | 1.26 | 1.96 | 46.70 | 35.87 | 43.24 | 15.71 |
| open | Ovre Forra | 2 | 1.24 | 1.76 | 47.06 | 43.80 | 44.28 | 29.07 |
| open | Ovre Forra | 3 | 1.23 | 1.74 | 46.97 | 44.10 | 44.61 | 29.61 |
| open | Ovre Forra | 4 | 1.21 | 1.70 | 46.91 | 44.70 | 45.31 | 30.68 |
| open | Ovre Forra | 5 | 1.23 | 1.74 | 47.23 | 44.16 | 44.80 | 29.71 |
| open | Ovre Forra | 6 | 1.28 | 1.74 | 47.01 | 44.15 | 42.72 | 29.70 |
| open | Ovre Forra | 7 | 1.17 | 1.73 | 47.50 | 44.25 | 47.50 | 29.87 |
| open | Ovre Forra | 8 | 1.27 | 1.74 | 46.97 | 44.10 | 43.39 | 29.61 |
| open | Ovre Forra | 9 | 1.30 | 1.76 | 46.70 | 43.88 | 41.91 | 29.20 |
| open | Ovre Forra | 10 | 1.21 | 1.73 | 46.91 | 44.33 | 45.17 | 30.01 |
| open | Ovre Forra | 11 | 1.24 | 1.75 | 47.05 | 44.01 | 44.50 | 29.45 |
| open | Ovre Forra | 12 | 1.30 | 1.76 | 46.86 | 43.88 | 42.05 | 29.20 |
| open | Midtfjellmosen | 1 | 1.20 |  | 45.00 |  | 43.75 |  |
| open | Ursee | 1 | 0.85 | 2.18 | 43.79 | 51.26 | 64.31 | 20.12 |
| open | Ursee | 2 | 0.77 | 2.06 | 43.72 | 42.43 | 48.45 | 17.63 |
| open | Ursee | 3 | 0.76 | 2.17 | 44.11 | 43.53 | 44.11 | 17.17 |
| open | Ursee | 4 | 0.76 | 1.85 | 44.11 | 47.77 | 59.28 | 22.09 |
| open | Ursee | 5 | 0.63 | 2.07 | 43.81 | 38.85 | 48.31 | 16.09 |
| open | Ursee | 6 | 0.69 | 2.21 | 45.12 | 44.31 | 55.79 | 17.23 |
| open | Ursee | 7 | 0.73 | 2.06 | 43.75 | 42.43 | 55.64 | 17.63 |
| open | Ursee | 8 | 0.61 | 2.01 | 43.71 | 45.65 | 61.34 | 19.63 |
| open | Ursee | 9 | 0.74 | 2.13 | 43.80 | 45.05 | 59.28 | 18.11 |
| open | Ursee | 10 | 0.69 | 2.19 | 45.12 | 43.92 | 55.79 | 17.20 |
| open | Ursee | 11 | 0.63 | 2.21 | 43.81 | 44.31 | 49.94 | 17.23 |
| open | Vejumbro | 1 | 1.00 | 2.60 | 43.27 | 43.15 | 50.41 | 19.34 |
| open | Vejumbro | 2 | 1.40 | 2.27 | 41.74 | 29.40 | 44.87 | 15.08 |
| open | Vejumbro | 3 | 1.26 | 2.35 | 46.87 | 35.96 | 43.57 | 17.70 |
| open | Vejumbro | 4 | 1.15 | 2.74 | 38.34 | 39.57 | 38.82 | 16.83 |
| open | Vejumbro | 5 | 1.15 | 2.96 | 41.60 | 29.29 | 42.36 | 11.54 |
| open | Vejumbro | 6 | 1.09 | 2.69 | 34.50 | 37.21 | 36.95 | 16.15 |
| open | Vejumbro | 7 | 1.76 | 2.09 | 46.84 | 28.78 | 31.12 | 16.06 |
| open | Vejumbro | 8 | 1.60 | 2.49 | 43.29 | 36.73 | 42.97 | 17.24 |
| open | Vejumbro | 9 | 1.21 | 2.69 | 39.25 | 37.21 | 37.95 | 16.15 |
| open | Vejumbro | 10 | 1.29 | 3.47 | 33.58 | 45.11 | 30.49 | 15.17 |
| ST | Lakkasuo | 1 | 0.64 | 1.13 | 45.01 | 47.23 | 62.42 | 37.92 |
| ST | Lakkasuo | 2 | 0.65 | 1.26 | 44.72 | 48.47 | 58.89 | 34.55 |
| ST | Lakkasuo | 3 | 0.66 | 1.66 | 42.53 | 48.42 | 56.53 | 25.07 |
| ST | Lakkasuo | 4 | 0.68 | 1.95 | 42.81 | 46.70 | 55.60 | 20.54 |
| ST | Lakkasuo | 5 | 0.65 | 1.13 | 44.08 | 48.12 | 60.50 | 38.47 |
| ST | Lakkasuo | 6 | 0.66 | 1.23 | 44.70 | 46.46 | 58.87 | 35.21 |
| ST | Lakkasuo | 7 | 0.73 | 1.40 | 42.89 | 43.38 | 51.38 | 27.00 |
| ST | Lakkasuo | 8 | 0.78 | 1.50 | 45.43 | 45.47 | 50.13 | 26.76 |
| ST | Lakkasuo | 9 | 0.69 | 1.27 | 44.15 | 47.18 | 57.51 | 31.82 |
| ST | Lakkasuo | 10 | 0.63 | 0.97 | 45.14 | 47.76 | 63.83 | 44.70 |
| ST | Lakkasuo | 11 | 0.65 | 1.19 | 43.55 | 46.87 | 58.50 | 36.37 |
| ST | Lakkasuo | 12 | 0.68 | 1.22 | 43.63 | 48.84 | 56.62 | 35.67 |
| ST | Lakkasuo | 13 | 0.74 | 1.48 | 42.01 | 46.54 | 49.89 | 27.53 |
| ST | Lakkasuo | 14 | 0.66 | 1.21 | 44.40 | 45.40 | 57.70 | 34.60 |
| ST | Lakkasuo | 15 | 0.62 | 1.10 | 44.82 | 46.40 | 61.87 | 38.83 |
| ST | Lakkasuo | 16 | 0.66 | 1.22 | 44.67 | 47.20 | 58.81 | 37.82 |
| ST | Lakkasuo | 17 | 0.69 | 1.51 | 42.25 | 48.41 | 54.50 | 29.41 |
| ST | Lakkasuo | 18 | 0.71 | 1.46 | 43.58 | 47.82 | 53.95 | 29.68 |
| ST | Lakkasuo | 19 | 0.66 | 1.27 | 43.97 | 45.61 | 58.19 | 34.13 |
| ST | Rospuda | 1 | 1.06 | 1.96 | 39.87 | 35.87 | 34.34 | 15.71 |
| ST | Rospuda | 2 | 1.11 | 1.96 | 40.90 | 35.87 | 33.66 | 15.71 |
| ST | Rospuda | 3 | 1.11 | 2.31 | 40.90 | 32.46 | 36.66 | 12.05 |
| ST | Rospuda | 4 | 1.16 | 2.31 | 41.92 | 32.46 | 36.98 | 12.05 |
| ST | Rospuda | 5 | 1.06 | 1.96 | 39.87 | 35.87 | 32.34 | 15.71 |
| ST | Rospuda | 6 | 1.16 | 2.13 | 41.92 | 34.16 | 33.98 | 13.88 |
| ST | Rospuda | 7 | 1.11 | 2.31 | 40.90 | 32.46 | 33.66 | 12.05 |
| ST | Rospuda | 8 | 1.06 | 1.96 | 39.87 | 35.87 | 34.34 | 15.71 |
| ST | Rospuda | 9 | 1.16 | 2.13 | 41.92 | 34.16 | 34.98 | 13.88 |
| ST | Rospuda | 10 | 1.06 | 2.31 | 39.87 | 32.46 | 32.34 | 12.05 |
| ST | Rospuda | 11 | 1.11 | 2.31 | 40.90 | 32.46 | 35.66 | 12.05 |
| ST | Rospuda | 12 | 1.06 | 1.96 | 39.87 | 35.87 | 32.34 | 15.71 |
| ST | Rospuda | 13 | 1.11 | 2.13 | 40.90 | 34.16 | 33.66 | 13.88 |
| ST | Rospuda | 14 | 1.06 | 2.13 | 39.87 | 34.16 | 32.34 | 13.88 |
| ST | Rotmeer | 1 | 0.40 | 0.70 | 38.56 | 46.58 | 82.61 | 47.42 |
| ST | Rotmeer | 2 | 0.57 | 0.78 | 43.38 | 46.65 | 65.65 | 51.43 |
| ST | Rotmeer | 3 | 0.61 | 0.74 | 42.74 | 44.31 | 59.90 | 51.16 |
| ST | Rotmeer | 4 | 0.47 | 0.76 | 42.39 | 46.34 | 77.08 | 52.03 |
| ST | Rotmeer | 5 | 0.67 | 0.81 | 42.69 | 45.61 | 59.50 | 51.67 |
| ST | Rotmeer | 6 | 0.51 | 0.88 | 43.12 | 46.65 | 72.61 | 52.00 |
| ST | Rotmeer | 7 | 0.40 | 0.76 | 38.56 | 46.34 | 82.61 | 52.03 |
| ST | Rotmeer | 8 | 0.89 | 1.14 | 42.74 | 47.81 | 41.27 | 36.01 |
| ST | Rotmeer | 9 | 0.61 | 0.94 | 42.74 | 43.14 | 59.90 | 39.25 |
| ST | Rotmeer | 10 | 0.57 | 0.94 | 43.38 | 43.14 | 65.65 | 39.25 |
| ST | Rotmeer | 11 | 0.47 | 0.70 | 42.39 | 46.58 | 77.08 | 47.42 |
| ST | Rotmeer | 12 | 0.89 | 0.90 | 42.74 | 45.26 | 41.27 | 43.35 |
| ST | Rotmeer | 13 | 0.89 | 1.14 | 42.74 | 47.81 | 41.27 | 36.01 |
| ST | Degerö | 1 | 0.59 | 0.94 | 43.14 | 45.87 | 66.00 | 50.53 |
| ST | Degerö | 2 | 0.65 | 0.97 | 45.19 | 45.97 | 60.39 | 43.49 |
| ST | Degerö | 3 | 0.77 | 1.03 | 43.58 | 46.21 | 51.69 | 40.81 |
| ST | Degerö | 4 | 0.78 | 0.97 | 45.53 | 46.61 | 51.88 | 43.14 |
| ST | Degerö | 5 | 0.52 | 0.87 | 42.82 | 46.07 | 71.55 | 51.49 |
| ST | Degerö | 6 | 0.61 | 0.75 | 42.97 | 45.21 | 62.98 | 52.07 |
| ST | Degerö | 7 | 0.79 | 1.05 | 44.91 | 46.67 | 48.45 | 42.16 |
| ST | Degerö | 8 | 0.83 | 0.92 | 43.37 | 45.63 | 49.74 | 42.60 |
| ST | Degerö | 9 | 0.77 | 0.84 | 44.20 | 46.00 | 50.24 | 47.35 |
| ST | Degerö | 10 | 0.50 | 0.69 | 42.88 | 45.23 | 74.55 | 52.59 |
| ST | Degerö | 11 | 0.70 | 0.80 | 43.03 | 45.84 | 53.35 | 49.15 |
| ST | Degerö | 12 | 0.85 | 1.17 | 43.69 | 46.87 | 45.96 | 39.87 |
| ST | Degerö | 13 | 0.79 | 1.00 | 44.91 | 46.08 | 48.45 | 40.60 |
| ST | Degerö | 14 | 0.79 | 0.90 | 43.48 | 46.55 | 53.17 | 45.65 |
| ST | Degerö | 15 | 0.50 | 0.68 | 42.73 | 45.16 | 75.21 | 46.58 |
| ST | Degerö | 16 | 0.58 | 0.73 | 43.43 | 44.64 | 66.36 | 55.23 |
| ST | Degerö | 17 | 0.65 | 0.81 | 43.44 | 45.57 | 65.75 | 48.52 |
| ST | Degerö | 18 | 0.61 | 0.96 | 43.08 | 45.75 | 60.44 | 43.16 |
| ST | Degerö | 19 | 0.67 | 0.76 | 43.88 | 45.08 | 60.93 | 50.90 |

Table S3: Relative abundance of molecules [%] for all sites, separated for marker classes

| **cover** | **site** | **sample_no** | **Vegetation** | | **Metabolized** | | **Primary vs Metabolized** | |
| --- | --- | --- | --- | --- | --- | --- | --- | --- |
|  |  |  | **pristine** | **drained** | **pristine** | **drained** | **pristine** | **drained** |
| open | Letterunshin | 1 | 54.74 | 5.50 | 11.17 | 22.56 | 4.90 | 0.24 |
| open | Letterunshin | 2 | 35.29 | 4.51 | 14.48 | 25.47 | 2.57 | 0.18 |
| open | Letterunshin | 3 | 48.00 | 5.06 | 12.14 | 23.68 | 3.96 | 0.21 |
| open | Letterunshin | 4 | 23.99 | 3.66 | 16.24 | 23.29 | 1.48 | 0.15 |
| open | Letterunshin | 5 | 53.89 | 5.88 | 11.51 | 24.25 | 4.77 | 0.25 |
| open | Letterunshin | 6 | 42.74 | 2.81 | 19.87 | 21.10 | 2.15 | 0.13 |
| open | Letterunshin | 7 | 47.34 | 4.18 | 10.35 | 25.36 | 4.74 | 0.16 |
| open | Letterunshin | 8 | 48.74 | 3.87 | 15.52 | 24.54 | 3.53 | 0.16 |
| open | Midtfjellmosen | 1 | 67.66 |  | 12.09 |  | 5.60 |  |
| open | Midtfjellmosen | 2 | 66.64 |  | 12.65 |  | 5.28 |  |
| open | Midtfjellmosen | 3 | 65.63 |  | 13.21 |  | 4.97 |  |
| open | Midtfjellmosen | 4 | 62.31 |  | 15.10 |  | 4.42 |  |
| open | Midtfjellmosen | 5 | 59.89 |  | 14.94 |  | 4.31 |  |
| open | Midtfjellmosen | 6 | 56.51 |  | 16.08 |  | 3.83 |  |
| open | Midtfjellmosen | 7 | 56.26 |  | 16.22 |  | 3.75 |  |
| open | Midtfjellmosen | 8 | 58.31 |  | 17.35 |  | 3.65 |  |
| open | Midtfjellmosen | 9 | 52.97 |  | 16.31 |  | 3.56 |  |
| open | Midtfjellmosen | 10 | 53.03 |  | 16.68 |  | 3.47 |  |
| open | Midtfjellmosen | 11 | 53.63 |  | 19.98 |  | 2.68 |  |
| open | Midtfjellmosen | 12 | 46.37 |  | 19.52 |  | 2.37 |  |
| open | Ovre Forra | 1 | 41.01 |  | 7.77 |  | 5.36 |  |
| open | Ovre Forra | 2 | 44.09 |  | 9.35 |  | 5.07 |  |
| open | Ovre Forra | 3 | 40.56 |  | 9.98 |  | 4.25 |  |
| open | Ovre Forra | 4 | 35.85 |  | 10.19 |  | 4.17 |  |
| open | Ovre Forra | 5 | 35.56 |  | 9.88 |  | 4.16 |  |
| open | Ovre Forra | 6 | 34.10 |  | 8.30 |  | 4.11 |  |
| open | Ovre Forra | 7 | 40.25 |  | 11.45 |  | 3.52 |  |
| open | Ovre Forra | 8 | 35.60 |  | 10.84 |  | 3.42 |  |
| open | Ovre Forra | 9 | 24.64 |  | 14.08 |  | 1.75 |  |
| open | Vejumbro | 1 | 15.78 | 9.87 | 11.25 | 20.00 | 1.40 | 0.49 |
| open | Vejumbro | 2 | 16.08 | 6.66 | 11.97 | 22.60 | 1.34 | 0.29 |
| open | Vejumbro | 3 | 14.95 | 7.70 | 10.81 | 21.13 | 1.38 | 0.36 |
| open | Vejumbro | 4 | 15.86 | 7.97 | 11.32 | 24.05 | 1.40 | 0.33 |
| open | Vejumbro | 5 | 15.98 | 8.70 | 11.97 | 18.96 | 1.34 | 0.46 |
| open | Vejumbro | 6 | 16.95 | 7.70 | 11.81 | 21.13 | 1.44 | 0.36 |
| open | Vejumbro | 7 | 15.84 | 7.51 | 11.32 | 24.30 | 1.40 | 0.31 |
| ST | Rospuda | 1 |  | 36.71 |  | 7.75 |  | 4.75 |
| ST | Rospuda | 2 |  | 40.87 |  | 7.61 |  | 5.37 |
| ST | Rospuda | 3 |  | 32.54 |  | 7.88 |  | 4.12 |
| ST | Rospuda | 4 |  | 37.71 |  | 7.86 |  | 4.81 |
| ST | Rospuda | 5 |  | 30.51 |  | 13.02 |  | 3.25 |
| ST | Rospuda | 6 |  | 28.99 |  | 12.63 |  | 3.08 |
| ST | Rospuda | 7 |  | 29.00 |  | 15.05 |  | 2.76 |
| ST | Rospuda | 8 |  | 31.94 |  | 7.74 |  | 4.13 |
| ST | Rospuda | 9 |  | 33.74 |  | 7.92 |  | 4.26 |
| ST | Rospuda | 10 |  | 30.18 |  | 13.14 |  | 3.21 |
| ST | Rospuda | 11 |  | 27.63 |  | 13.90 |  | 2.78 |
| ST | Rospuda | 12 |  | 32.56 |  | 11.39 |  | 3.72 |
| ST | Rospuda | 13 |  | 25.42 |  | 14.95 |  | 2.35 |

Table S4: Variable loadings on the first two principal components for (a) all peatland sites, (b) open peatlands, and (c) sparsely treed peatlands. Positive and negative values indicate the direction of association with each principal component, while larger absolute values indicate a stronger contribution to the respective axis.

| **PCA 1** |  | |  | |  | | **PCA 2** | |  | | |  | |  | | |  |
| --- | --- | --- | --- | --- | --- | --- | --- | --- | --- | --- | --- | --- | --- | --- | --- | --- | --- |
|  | |  | |  | |  | |  | | sparsely treed | | | open | | |  |  |
| **Variable** | | **PC1** | | **PC2** | |  | | Variable | | PC1 | PC2 | | PC1 | | PC2 |  |  |
| NDVI | | -0.365 | | 0.652 | |  | | NDVI | | 0.291 | -0.512 | | 0.365 | | 0.308 |  |  |
| GI | | -0.509 | | 0.314 | |  | | GI | | 0.455 | -0.102 | | 0.412 | | 0.1 |  |  |
| N | | -0.497 | | -0.252 | |  | | N | | 0.459 | 0.08 | | 0.388 | | -0.182 |  |  |
| C | | 0.317 | | 0.626 | |  | | C | | -0.301 | -0.403 | | -0.195 | | 0.66 |  |  |
| CN | | 0.511 | | 0.146 | |  | | CN | | -0.439 | -0.122 | | -0.394 | | 0.108 |  |  |
|  | |  | |  | |  | | EVI | | 0.143 | 0.587 | | 0.413 | | 0.128 |  |  |
|  | |  | |  | |  | | gNDVI | | 0.116 | -0.429 | | 0.255 | | 0.551 |  |  |
|  | |  | |  | |  | | Albedo | | -0.424 | 0.118 | | 0.343 | | -0.307 |  |  |

Figure S1: Conceptual illustration of the sampling design in pristine and drained peatland areas. The grid represents the 3 × 3 m PlanetScope pixels, while red stars indicate the locations of three replicate peat cores collected within each hydrological condition. Spectral indices were averaged across the delineated peatland polygons, whereas biogeochemical measurements were used to characterize the sampled field plots. The illustration is schematic and not scale.

Figure S2: (a) NDVI and (b) GI in pristine and drained sites in early summer 2023 for all investigated sites

Figure S3: (a) EVI and (b) Albedo, separated for cover types (i) open and (ii) sparsely treed, in pristine and drained sites in early summer 2023 for all investigated site

Figure S4: Time series of NDVI values across all open peatland sites, distinguished between pristine and drained conditions

Figure S5: Time series of GI values across all (a) open and (b) sparsely treed peatland sites, distinguished between pristine and drained conditions

 Figure S6: Time series of EVI values across all (a) open and (b) sparsely treed peatland sites, distinguished between pristine and drained conditions

Figure S7: Time series of albedo values across all (a) open and (b) sparsely treed peatland sites, distinguished between pristine and drained conditions
